# Automatic analysis and 3D-modelling of Hi-C data using TADbit reveals structural features of the fly chromatin colors

**DOI:** 10.1371/journal.pcbi.1005665

**Published:** 2017-07-19

**Authors:** François Serra, Davide Baù, Mike Goodstadt, David Castillo, Guillaume J. Filion, Marc A. Marti-Renom

**Affiliations:** 1 CNAG-CRG, Centre for Genomic Regulation (CRG), Barcelona Institute of Science and Technology (BIST), Barcelona, Spain; 2 Gene Regulation, Stem Cells and Cancer Program, Centre for Genomic Regulation (CRG), Barcelona, Spain; 3 Universitat Pompeu Fabra (UPF), Barcelona, Spain; 4 ICREA, Barcelona, Spain; UCSD, UNITED STATES

## Abstract

The sequence of a genome is insufficient to understand all genomic processes carried out in the cell nucleus. To achieve this, the knowledge of its three-dimensional architecture is necessary. Advances in genomic technologies and the development of new analytical methods, such as Chromosome Conformation Capture (3C) and its derivatives, provide unprecedented insights in the spatial organization of genomes. Here we present TADbit, a computational framework to analyze and model the chromatin fiber in three dimensions. Our package takes as input the sequencing reads of 3C-based experiments and performs the following main tasks: (i) pre-process the reads, (ii) map the reads to a reference genome, (iii) filter and normalize the interaction data, (iv) analyze the resulting interaction matrices, (v) build 3D models of selected genomic domains, and (vi) analyze the resulting models to characterize their structural properties. To illustrate the use of TADbit, we automatically modeled 50 genomic domains from the fly genome revealing differential structural features of the previously defined chromatin colors, establishing a link between the conformation of the genome and the local chromatin composition. TADbit provides three-dimensional models built from 3C-based experiments, which are ready for visualization and for characterizing their relation to gene expression and epigenetic states. TADbit is an open-source Python library available for download from https://github.com/3DGenomes/tadbit.

This is a *PLOS Computational Biology* Software paper.

## Introduction

**Fig 1:**
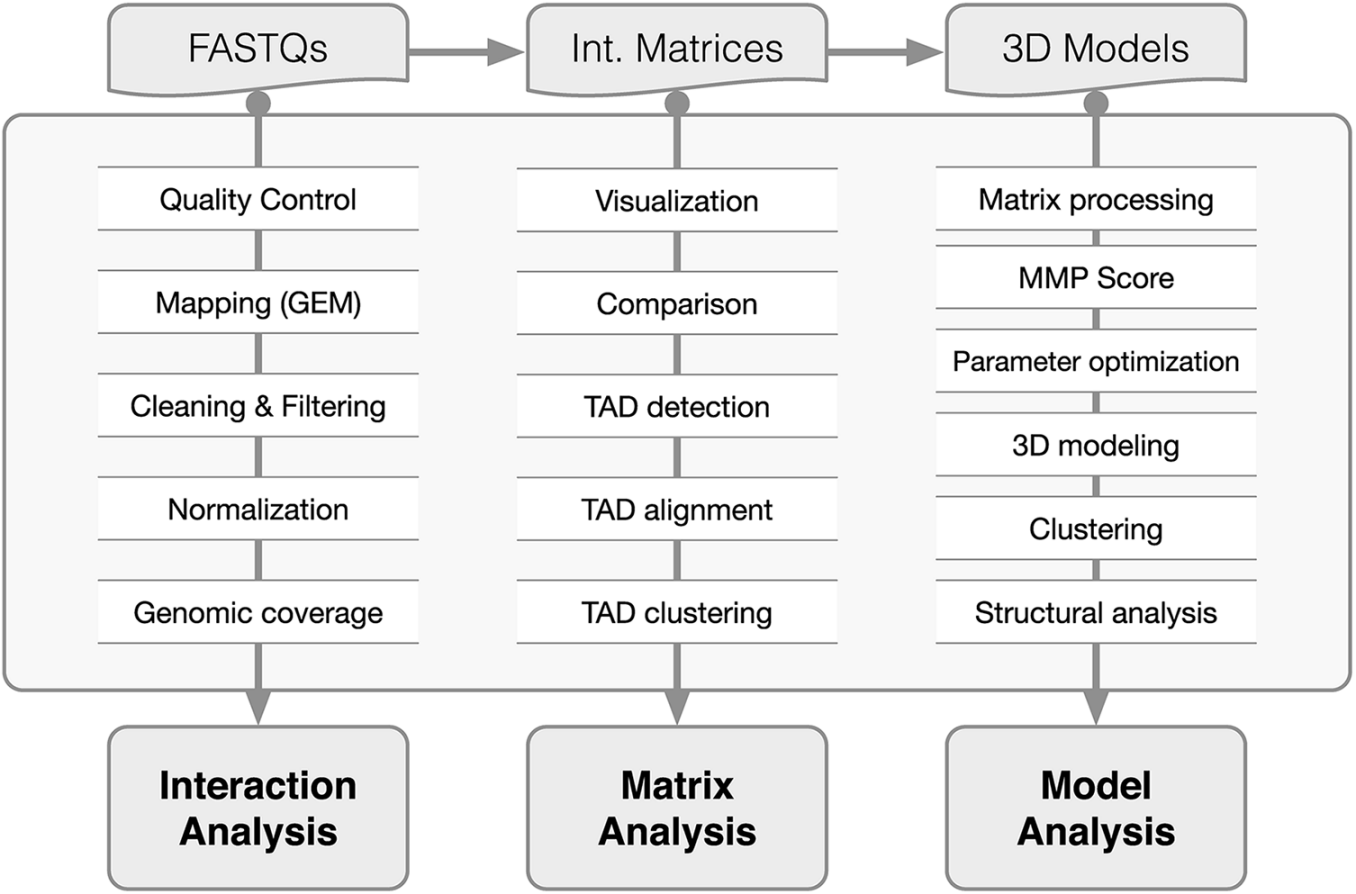
TADbit flowchart. Main functions of the TADbit library from FASTQ files to 3D model analysis. TADbit accepts many input data types such as FASTQ files, interaction matrices and 3D models. A series of python functions in TADbit (**Supplementary Text**) allow for the full analysis of the interaction data, interaction matrices as well as derived 3D models.

Metazoan genomes are organized within the cell nucleus. At the highest level, chromosomes occupy characteristic nuclear areas or “chromosome territories”, separated by inter-chromatin compartments [1]. Underneath, chromosomes have additional levels of arrangements and organize themselves into the A and B compartments [2], which in turn are composed of Topologically Associating Domains (TADs), defined as regions of the DNA with a high frequency of self-interactions [3–5]. Determining the three-dimensional (3D) organization of such genomic domains is essential for characterizing how genes and their regulatory elements arrange in space to carry out their functions [6]. Chromosome Conformation Capture (3C) [7] and its derived methods (here referred to as 3C-based methods) are now widely used to elucidate the spatial arrangement of genomes [8]. Although the frequency of interactions between loci can be used as a proxy for their spatial proximity, 3C-based contact maps do not easily convey all the information about the spatial organization of a chromosome. This information, however, can be inferred using computational methods [9]. Here we present TADbit, a Python library for the analysis and 3D modeling of 3C-based data. TADbit takes as input the sequencing reads of 3C-based experiments and performs the following main tasks: (i) pre-process the reads, (ii) map the reads to a reference genome, (iii) filter and normalize the interaction data, (iv) analyze the resulting interaction matrices, (v) build 3D models of selected genomic domains, and (vi) analyze the resulting models to characterize their structural properties (Fig 1). TADbit builds on existing partial implementations of methods for 3D genomic reconstruction [10–20]. As a validation of the model-building module of TADbit, a systematic analysis of its limitations has shown that 3D reconstruction of genomes based on 3C-based data can produce accurate 3D models [21].

TADbit has been already shown to provide biological insights [22–24]. Here, we introduce a new application of TADbit for the modeling and analysis of 50 genomic domains of the *Drosophila melanogaster* genome. It was shown that the *Drosophila* genome consists of five distinct chromatin types determined by mapping 53 broadly selected chromatin proteins and four key histone modifications [25]. The chromatin types were labeled with colors and comprise “blue” chromatin, enriched in Polycomb group proteins and H3K27 methylation, “green” chromatin, bound by HP1 and located at peri-centromeric regions, “yellow” and “red” chromatin, harboring distinct classes of active genes, and “black” chromatin, covering more than 40% of the *Drosophila* genome and characterized by low occupancy of most chromatin markers. More recently, genome-wide 3C-based interaction maps in *Drosophila* revealed that TAD boundaries are gene-dense, highly bound by transcription factors and insulator proteins and correspond to transcribed regions [5, 26]. Moreover, it was shown that the active red and yellow chromatin types preferentially locate at TAD borders while the others preferentially locate inside TADs. This work highlighted the existence of interplay between the structural organization of genomic domains and their chromatin composition. Similar relationships have also been observed in other organisms, including mouse and human [27–30].

To further characterize the structural properties of the *Drosophila* chromatin types, we have used TADbit on available Hi-C data. By building 3D models of genomic domains covering more than 50 Mb of the *Drosophila* genome, we show that the five previously described chromatin colors are characterized by distinct structural properties. Black chromatin is a compact, dense and closed chromatin fiber. In comparison, the heterochromatic types blue and green are more open and accessible. Finally, the yellow and red types feature a loose and open chromatin, potentially accessible to proteins and transcription factors responsible for regulating resident genes.

## Design and implementation

TADbit has been implemented as a Python library to deal with all steps to analyze, model and explore 3C-based data. With TADbit the user can map FASTQ files to obtain raw interaction binned matrices (Hi-C like matrices), normalize and correct interaction matrices, identify and compare Topologically Associating Domains (TADs), build 3D models from the interaction matrices, and finally, extract structural properties from the models. Next, we describe in more details each of the main independent tasks that can be executed with TADbit:

### FASTQ quality check

The TADbit pipeline starts by performing a quality control on the raw data in FASTQ format. This quality check is similar to the tests performed by the *FastQC* program [31] with adaptions for Hi-C datasets (S1 Fig).

### Iterative mapping

TADbit implements an iterative mapping strategy that is a slightly modified version of the original ICE method developed for the HiClib library [32]. The minimal differences with the original ICE method are the mapper used (TADbit uses GEM [33]) and a more flexible way to define the position of the iterative mapping windows, which can now be fully defined by the user.

### Fragment based filtering

The filtering strategy implemented in TADbit builds on previously described protocols [32] to correct all the computationally detectable experimental biases/errors. After mapping, TADbit can filter the reads depending on ten criteria (S2 Fig), which can be applied individually or as a set of filters.

### Interaction matrix cleaning and normalization

Once filtered, the read-pairs are binned at a user-specified resolution (bin size) depending on the average density per cell required by the analysis to be performed. However, a minimum amount of counts per bin is usually required for the normalization of the data [32]. To determine the threshold amount of interactions for masking columns, TADbit proceeds in two steps. First, the columns with zero counts are removed. Second, a polynomial is fitted to the empirical distribution of the total amount of interactions per column, and the first mode of this distribution is used to define the exclusion threshold value below which columns will be removed. After the column removal, the remaining bins are further normalized to remove local genomic biases (*e*.*g*., to correct for the genomic regions with higher mappability and/or PCR amplification). The normalization procedure implemented in TADbit is a modification of the ICE balancing method implemented in the HiClib library [32]. The modification in TADbit consists simply in truncating the balancing process of the ICE normalization after an undefined number of iterations. In TADbit, and with default parameters, the ICE normalization stops when a maximum of 10% of variability between the sum of interactions in a given bin and the average over the genomic matrix is reached (this percentage of variability can be user-defined).

### Comparison of interaction matrices

**Fig 2:**
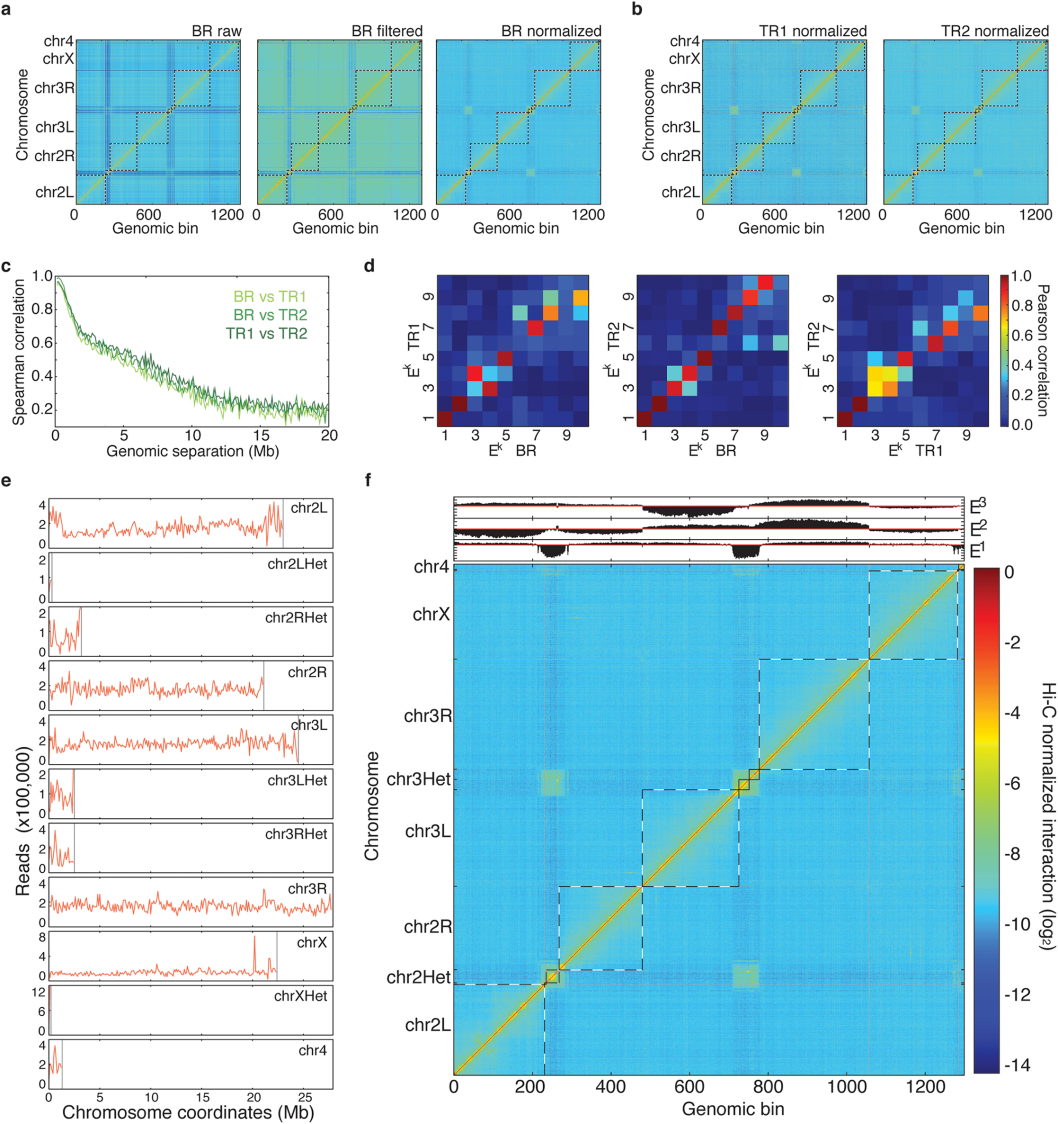
Hi-C interaction maps at 100 kb resolution for the entire *Drosophila* genome. (a) Raw, filtered and normalized genome-wide interaction maps for the BR dataset. Only after the normalization of the data, the enriched interaction between centromere regions of the *Drosophila* chromosomes can be observed. (b) Normalized maps for the TR1 and TR2 datasets. (c) Comparison of the normalized Hi-C maps between the three datasets at 100 kb resolution. The Spearman correlation was computed between off-diagonal regions as a function of their genomic distance. (d) Matrices of Pearson correlation coefficients of main eigenvectors from the three Hi-C datasets (that is, BR, TR1 and TR2). The data shows the expected high correlation of the top three eigenvectors [32]. (e) Genomic coverage of the mapped reads per chromosome from the SUM dataset. (f) Hi-C normalized interaction matrix at 100 kb resolution for the SUM dataset. The three main eigenvectors of the normalized interaction matrix mark the position of centromeres (E^1^), chromosomes (E^2^), and chromosome arms (E^3^). TADbit automatically generated all the plots in the figure.

Once normalized, the Hi-C contact matrices can be compared to estimate their degree of similarity. For this purpose, TADbit implements plotting functions (S1 Text) and two comparison scores: (i) a Spearman rank correlation between bins in two matrices at increasing genomic distances (Fig 2C) and (ii) a Pearson correlation between the first eigenvectors of each matrix (Fig 2D). Although both measures aim at identifying whether two matrices are similar or not, they have different properties. The first one is sensitive to the matrix resolution and decays as the genomic distance of the compared bins increases. The second one provides a more global comparison of the matrices and aims at identifying whether the internal correlations in the matrix (detected by its principal eigenvectors) are similar between the compared matrices.

### Genome segmentation into Topologically Associating Domains (TADs)

TADbit analyzes the contact distribution along the genome and subsequently segments it into its constitutive TADs, with each TAD border corresponding to a vertical slice of the Hi-C interaction matrix. TADs can be computed on the interaction matrix from a single experiment or from the matrix resulting from the merge of different experiments. To calculate the position of borders between TADs along a chromosome, TADbit employs a breakpoint detection algorithm [22] that returns the optimal segmentation of the chromosome under BIC-penalized likelihood (S2 Text). The algorithm in TADbit for segmenting the genome into TADs among others have been recently assessed [34].

### Alignment of TAD boundaries

TAD borders are conserved across different cell types and even across species, indicating that topological domains may play an important role in the organization of chromatin in metazoan genomes [3]. To assess whether TAD borders are conserved throughout different experiments, we implemented a multiple-experiment border alignment algorithm. Starting from different border definitions of the same genomic region, TADbit aligns each TAD to a consensus TAD list, either using the classic Needleman-Wunsch algorithm [35] or using a method based on reciprocal closest boundaries.

### Three-dimensional (3D) modeling of genomic domains

In TADbit, the three-dimensional (3D) models of selected genomic domains are generated by transforming the input 3C-based interaction maps into a set of spatial restraints that are later satisfied using the Integrative Modeling Platform (IMP) [36], as previously described [12].

### Structural clustering of the resulting 3D models

To assess the structural similarity of the generated models, TADbit first structurally aligns them using a pair-wise rigid-body superposition that minimizes the Root Mean Squared Deviation (RMSD) between the superimposed conformations [37]. Then, a matrix with an all-against-all similarity score (S3 Text) is input in the Markov Cluster Algorithm (MCL) program [38] for generating unsupervised sets of clusters of structurally related models.

### Structural analysis of the resulting 3D models

In this work, we have showed how TADbit could be used to model the 3D architecture of chromatin. However, we have implemented a detailed description of how to use each function implemented in TADbit and a series of structural analysis in TADbit to be applied on the generated 3D models (see online documentation and tutorials http://3dgenomes.github.io/TADbit) and outputs several measures to describe the architecture of the model.

### Output and visualization of 3D models

TADbit includes a simple three-dimensional model viewer using matplotlib [39], it is designed to be compatible with other visualizing tools, including TADkit (http://www.3DGenomes.org/TADkit).

## Results

### Chromatin interaction maps of the *Drosophila melanogaster* genome

The TADbit pipeline starts from raw data (*i*.*e*. reads generated from a 3C-based experiment). We downloaded SRA files from the NCBI Gene Expression Omnibus under accession number GSE38468 [26], and converted them to FASTQ files using the SRA Toolkit [39]. The dataset contained three separate Hi-C experiments [2] performed on *Drosophila* Kc167 cells using the restriction endonuclease HindIII, consisting of one biological replicate (SRR398921) and two technical replicates (SRR398318 and SRR398920), labeled here as “BR”, “TR1” and “TR2”. They comprised about 194, 67 and 112 million paired-end reads, respectively (Table 1). A quality check of the first million reads in each of the FASTQ file showed that the average PHRED scores [40] were higher than 25 across each of the 2x50 bp paired-end reads, which is indicative of good quality. Moreover, TADbit assessed that more than 95% of the reads had undergone digestion during the Hi-C experiment and only ~2% of the reads contained dangling ends *sensu stricto* (reads starting with a digested restriction site, S2 Fig). Next, the paired-end reads were aligned in TADbit to the *Drosophila* reference genome (dm3) using the GEM mapper [33] with a previously proposed iterative mapping strategy [32]. With this strategy, 67.0% to 77.8% of the original reads could be uniquely mapped (Table 1). After discarding those with only one mapped end, the number of mapped pairs diminished (50.2% to 63.5% of the original reads). These numbers were similar to those reported in the original experiments [26]. After mapping, the reads were further filtered as previously described [32], resulting in about 48, 24, and 41 million valid pairs (or interactions) for the BR, TR1 and TR2 experiments, respectively (Table 1). Finally, the filtered interaction maps were normalized using the iterative correction and eigenvector decomposition (ICE) procedure [32], also implemented in TADbit (Fig 2A). The resulting interaction matrices were highly correlated (Fig 2B, 2C and 2D), which prompted us to merge the input reads into a single dataset of more than 372 million reads. The new dataset, herein referred to as “SUM”, was also automatically filtered and normalized by TADbit (Fig 2E and 2F). The interaction map from the SUM dataset shows all the previously described features of the 3D organization of the *Drosophila* genome, including the chromosome arm territories, the clustering of centromeres and the infrequent interactions between telomeres.

**Table 1:** TADbit mapping and filtering of the Hi-C experimental results.

Sample (and read)	Exp.	Reads	Mapped reads	%	Mapped pairs	%	Valid pairs	%	Self circle		Dangling ends		Duplicates		Error		Close to RE		Over represented.		Size	
**SRR398921 read 1**	BR	193,950,826	135,851,098	70.0%																		
**SRR398921 read 2**		193,950,826	129,852,420	67.0%	97,455,453	50.2%	48,213,546	24.9%	4,654,288	5%	18,838,598	19%	34,244,134	35%	3,567,709	4%	10,574,894	11%	3,530,086	4%	419,260	0%
**SRR398318 read 1**	TR1	67,206,936	52,292,246	77.8%																		
**SRR398318 read 2**		67,206,936	50,486,087	75.1%	42,653,629	63.5%	23,543,775	35.0%	2,969,872	7%	4,802,745	11%	8,255,524	19%	7,215,611	17%	2,319,417	5%	1,168,467	3%	92,402	0%
**SRR398920 read 1**	TR2	111,531,689	80,772,907	72.4%																		
**SRR398920 read 2**		111,531,689	81,942,023	73.5%	63,746,200	57.2%	40,863,217	36.6%	3,997,101	6%	6,778,442	11%	10,228,267	16%	5,775,673	9%	3,480,769	5%	1,666,146	3%	130,963	0%
**SRR398 read 1**	SUM	372,689,451	268,981,000	72.2%																		
**SRR398 read 2**		372,689,451	261,858,738	70.3%	203,816,546	54.7%	112,166,621	30.1%	11,857,601	6%	30,892,279	15%	54,272,206	27%	16,192,909	8%	16,617,598	8%	6,222,190	3%	648,110	0%

Columns indicate the number of reads per experiment (“Reads”), those that were uniquely mapped (“Mapped reads”), pairs of reads mapped in both ends (“Mapped pairs”) and finally the number of reads that remained after filtering (“Valid pairs”). The filtered reads were quantified by “Self circles”, “Dangling ends”, “Duplicates”, “Close to RE”, “Over represented”, and “Size” filters.

### The *Drosophila* genome is partitioned into TADs of different robustness

**Fig 3:**
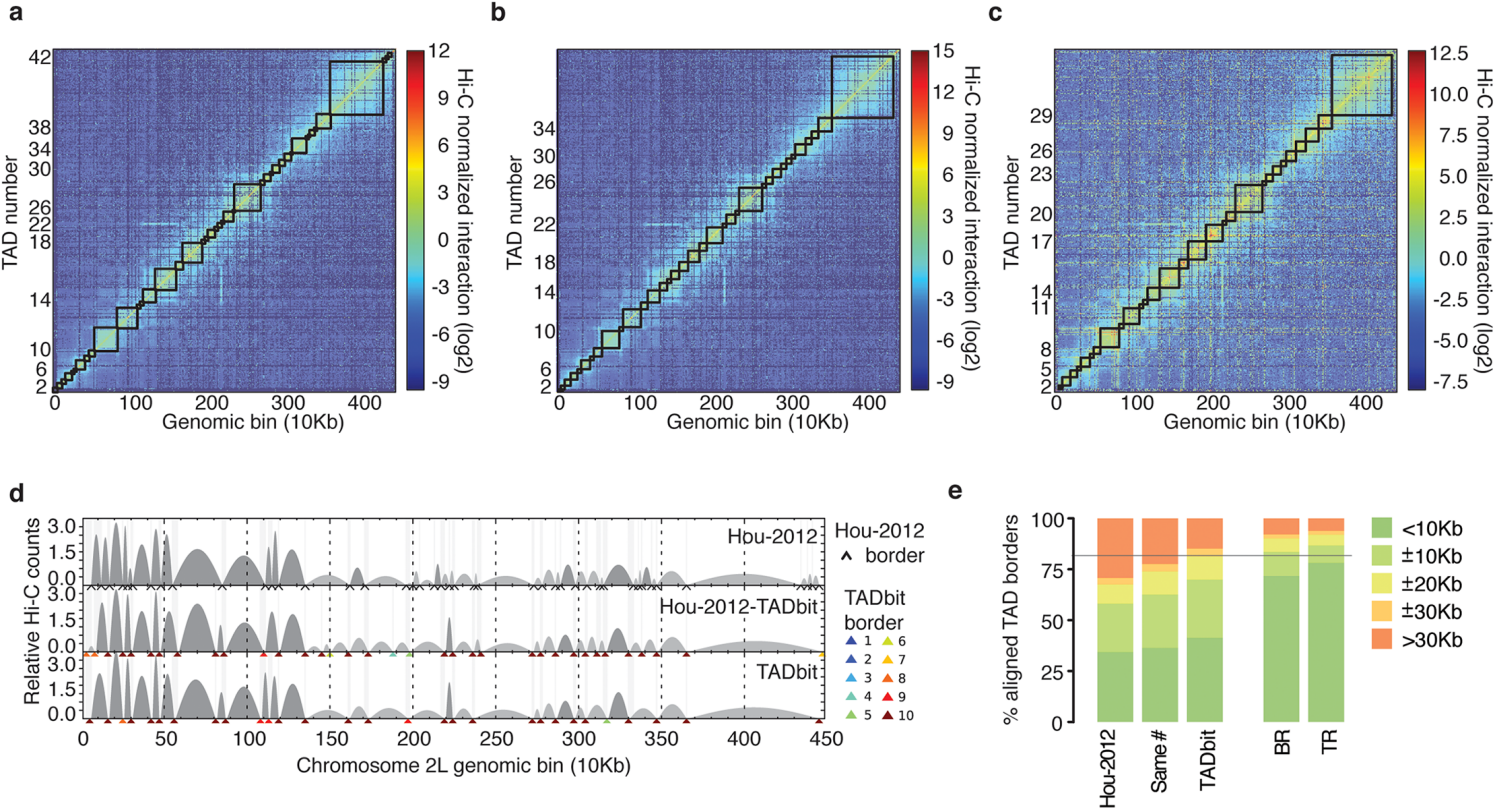
TAD border detection and comparison with the results from Hou *et al*. [26]. (a) Hi-C normalized interaction matrix at 10 kb resolution for the first 4.5 Mb of chromosome 2L in the *Drosophila* genome. Interactions matrix and TAD borders were obtained from published data [26]. (b) Hi-C normalized interaction matrix from the same genomic region and resolution as in panel a. The interaction counts are as previously published [26] but the TAD borders are those defined by TADbit. (c) Hi-C normalized interaction matrix from the same genomic region and resolution as in panel a. Interaction data and TAD borders are both generated by TADbit. (d) TAD border alignments between the three differently processed experimental data: borders defined in Hou *et al*. [26] (Hou-2012, top graph), borders defined by TADbit using the Hou-2012 matrix (mid graph), and borders and matrix determined by TADbit (bottom graph). Dark and light grey arches indicate TADs with higher and lower than expected intra-TAD interactions, respectively. TAD borders are indicated with a black arrow for the Hou-2012 defined borders and by color arrows for the TADbit identified borders. TADbit border robustness (from 1 to 10) is identified by a color gradient from blue to red. (e) Comparison of the agreement between the aligned TAD borders in the three datasets. As a reference, the horizontal grey line indicates a ±20 kb (2 bins) agreement between the biological replica (BR) and the first technical replicate (TR1) as determined by TADbit. The plots in panels *a* to *d* were automatically generated by TADbit.

Next, we generated 10 kb resolution interaction maps of the *Drosophila* genome to which we applied a TAD boundary detection algorithm implemented in TADbit (Design and Implementation and S2 Text). This algorithm uses a change-point detection approach inspired from methods used to identify copy number variations in CGH experiments [41]. Briefly, we use Poisson regression to find the most likely segmentation of the chromosome in *m* TADs and choose the value of *m* associated with the optimal Bayesian Information Criterion. In addition to the optimality of the solution, the main advantage of the new algorithm is the assignment of a robustness score to each TAD boundary (Design and Implementation and S2 Text). TADbit identified a total of 689 TADs with an average length of 162.8 kb (ranging from 20 kb to 1.5 Mb), representing larger TADs than previously reported [26]. Given the hierarchical organization of the genome [8], we set out to assess whether the difference was due to the identification of new borders or to the merging of the identified TADs. We downloaded the interaction matrices and the TAD borders as defined by Hou *et al*. [26] (here referred to as the original definition) and compared them to the borders obtained by running TADbit on these interaction matrices (Fig 3A, 3B and 3C). To this end we used the TADbit module to align multiple TAD boundaries from several experiments (Design and Implementation and Fig 3D). Overall, 81% of the borders defined by TADbit align within 20 kb of an original border when using the TADbit definition as reference (Fig 3E). The number decreases to 67% of the borders when using the original definition as a reference. By forcing TADbit to identify the same number of borders as the original definition (1,110 borders), the agreement increases to 74% within 20 kb. For comparison, the agreement of the TADbit border definitions between the three independent Hi-C experiments (BR, TR1 and TR2) is about 90%. The degree of similarity between the original and the TADbit definitions points to a variation of the algorithm sensitivity more than to discrepancies (see Fig 3D for instance). Moreover, the borders present only in the TADbit definition usually have a weak strength. Indeed, the agreement increases to 94% by comparing borders of 6 or higher strength as defined by TADbit. In summary, our results using TADbit confirm the previously described TAD level partitioning of the *Drosophila* genome and refine it with a confidence score. Such strength score could later be used to characterize the hierarchical organization of the genome in TADs or as an indicator of the confidence in the prediction (S3 Fig).

### Automatic modeling of 50 genomic regions of the *Drosophila* genome

**Fig 4:**
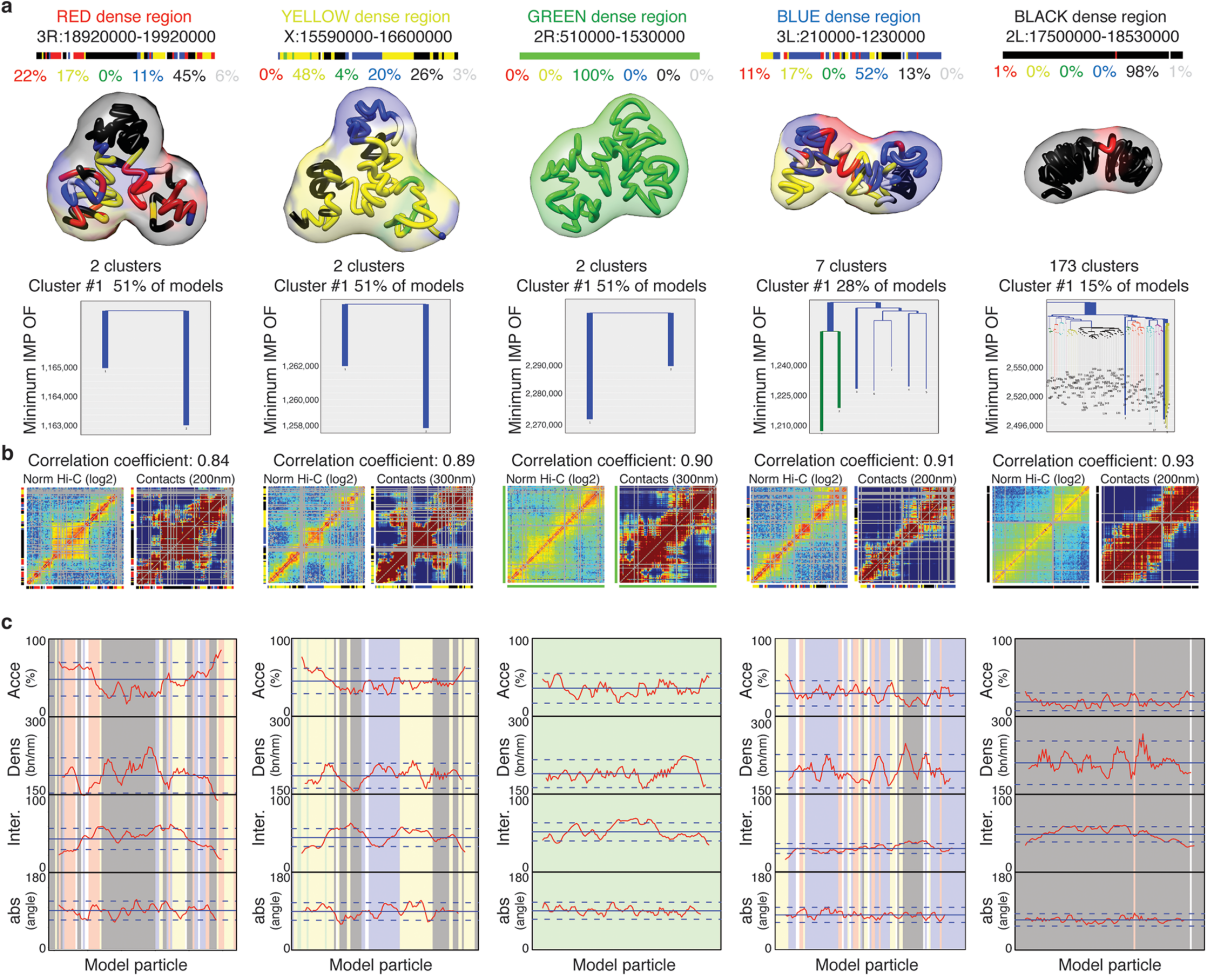
TADbit 3D models and structural properties. (a) Genomic coordinates, chromatin color proportions, 3D models and structural clustering for the five regions with highest coverage for each color in the *Drosophila* genome. The ensemble of models for cluster number 1 (the most populated cluster) for each color is represented by its centroid as a solid tube colored by its particle colors. The ensemble around the centroid is simulated by a transparent surface covering a Gaussian smooth surface 150 nm away from the centroid. Figures of 3D models were produced by Chimera [47]. The structural clustering of the 2,000 models produced per region were aligned with TADbit and clustered by structural similarity. Most modeled regions segregate into two large clusters corresponding to mirror images of each other. (b) Comparison of the input interaction Hi-C matrix to a contact map from the 2,000 built models per region, with Spearman correlation coefficient. (c) Structural properties by particle are shown for accessibility (percentage), density (bp per nanometer), interactions (number), and angle (degree). The background of the plot represents the color assigned to each of the particles in the models. TADbit automatically generated all plots.

Next, we used TADbit to model the 3D structure of 50 selected genomic regions of about 1 Mb each (S1 Table). It is important to note that there is not an optimal size for modeling a chromatin region. The optimal size depends on the experimental design, the underlying biological question and the computational power. The 50 regions were selected based on their chromatin colors composition [25]. The selection included the top ten regions of the genome most enriched in each of the five defined chromatin colors. Given the non-homogenous distribution of chromatin colors in the *Drosophila* genome, where the genome is composed of large stretches of black chromatin interspersed by shorter domains of blue, yellow and red chromatin (green chromatin is an exception, as it is mainly found in peri-centromeric regions and on chromosome 4), finding continuous 1 Mb stretches of chromatin for the blue, yellow and red colors was not always possible (Fig 4A). For instance, the highest red coverage in a 1 Mb region of the genome was only 22%. For yellow and blue, the maximum coverage was 48% and 52%, respectively, whereas for black and green chromatin types the maximum coverage was 98% and 100%, respectively.

All the selected genomic domains yielded a Matrix Modeling Potential (MMP) score [21] ranging from 0.85 to 0.96, which is predictive of high accuracy models (S1 Table). To model the 3D structure of the 50 regions, we used as input the Hi-C interaction matrix where each 10 kb bin was represented as a spherical particle in the model. All the particles were restrained in space based solely on their measured interactions, chain connectivity and excluded volume.

The size of the spherical particles representing 10 kb was defined by the relationship 0.01nm/bp assuming the canonical 30 nm fiber [42]. However, this relationship can be modified or optimized using the “scale” parameter in TADbit [12]. We modeled the chromatin as a homopolymer, assuming that the space occupied by each 10 kb piece of chromatin is constant. This strategy is necessary because of two reasons. First, the amount of free parameters needed to optimize the size of each particle independently is intractable statistically and computationally. And, second, we cannot define categories of particles using information about their epigenetic state (with for example smaller heterochromatic particles), because the information about epigenetic states of the chromatin is later used to assess the quality of the 3D models.

The conversion from interactions to distances was previously published [12]. Briefly, TADbit considers an inverse relationship between spatial distances and the corresponding frequencies of interactions. Given this assumption, TADbit transforms the frequencies of interactions into spatial restraints differently for consecutive and non-consecutive particles. Two consecutive particles are spatially restrained according to their occupancy, which corresponds to the sum of their radii. Non-consecutive particles are restrained based on empirically identified parameters that define a set of restraints, their distances and the forces applied to them. TADbit empirically identifies three optimal parameters using a grid search where a limited number of models are built for each set of parameters. The three parameters are: the proximal distance between two non-interacting particles, a lower bound cut-off to define particles that do not interact frequently and an upper bound cut-off defining particles that do interact frequently. Finally, the modeling parameters are optimized by maximizing the correlation between the contact map of the models and the input Hi-C interaction matrix (Design and Implementation and S1 Table).

All the 50 modeling exercises resulted in high correlations between the contact maps and the Hi-C interaction matrices, ranging from 0.83 to 0.93 (Fig 4B and S1 Table). Altogether, the modeled regions covered a total of 51.8 Mb of the *Drosophila* genome, forming the largest dataset of genomic regions modeled at 10 kb resolution (S4 Fig).

### Structural properties of the *Drosophila* chromatin colors

The generated models were automatically analyzed by TADbit to further characterize their structural properties. In particular, among the set of descriptive measures available in TADbit, we calculated four main structural properties for each particle (genomic bin) in the models. Those included: (i) *accessibility*, measuring how accessible from the outside a particle is; (ii) *density*, measuring the local compactness of the chromatin fiber; (iii) *interactions*, counting the number of particles within a given spatial distance from a selected particle; and (iv) *angle*, measuring the angle formed by a particle and its two immediate neighbor particles. To assess whether the different occupancy of proteins and chromatin modifications defining the five colors of chromatin had an influence on the 3D structure of the genome, we assigned to each particle one of the five chromatin colors if at least 50% of the 10 kb region was covered by this chromatin type [25]. Particles with non-homogenous colors were assigned to the undefined “white” color. These four measures provided an overview of the structural properties of each color in a particle-based manner. Models with decreasing amount of black, blue and green particles resulted in less compact and regular structures compared to those enriched in blue or black particles (Fig 4C). For example, the top black region (98% black, 1% red and 1% white) had low accessibility throughout, combined with a relatively high density (interestingly, the lowest density for that region corresponds to the only red particle), high number of interactions and closed angle between particles (Fig 4C last column).

**Fig 5:**
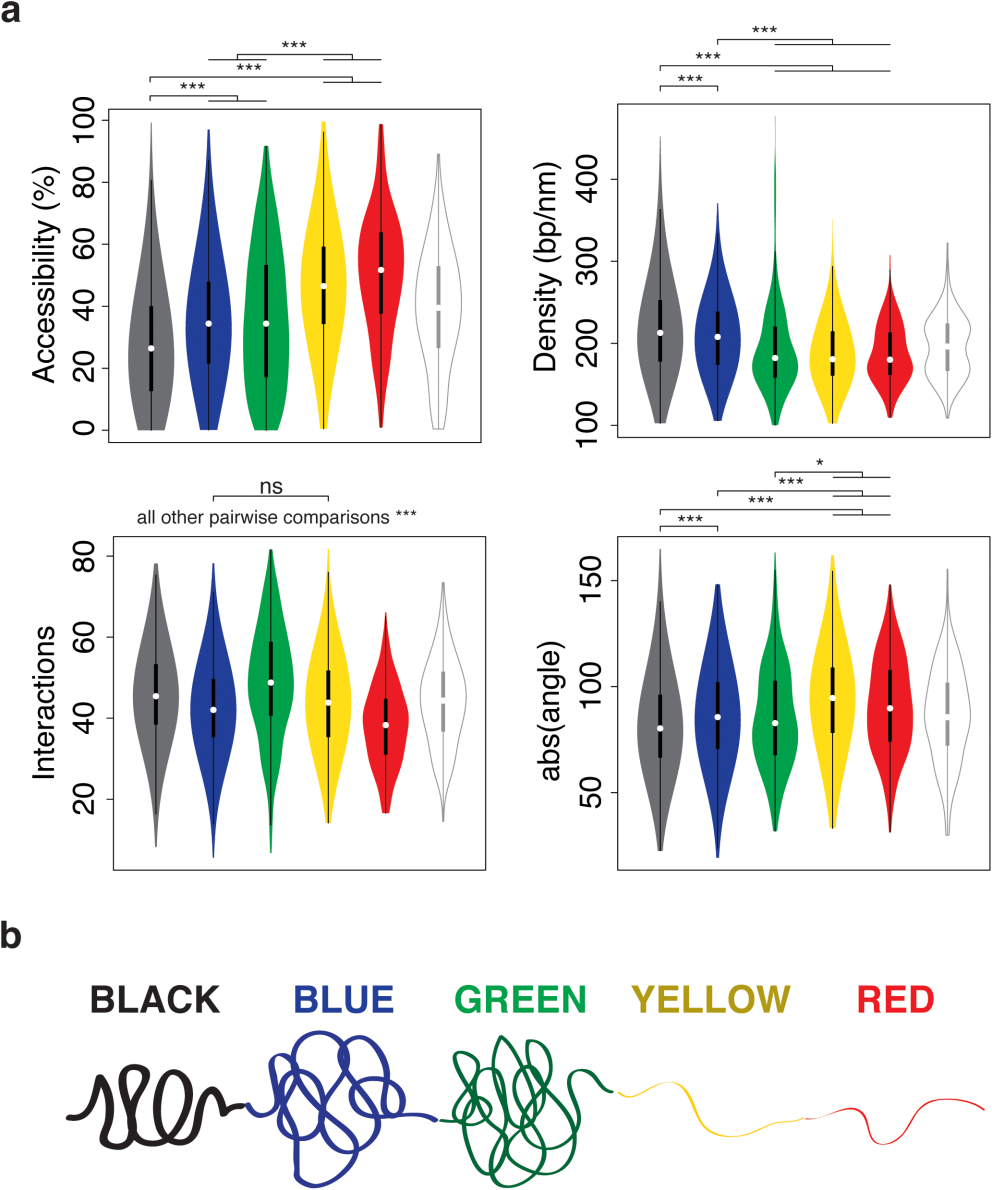
Structural properties of the five described chromatin colors. (a) Distribution of each of the four structural properties (that is, accessibility, density, interactions, and angle) grouped by chromatin colors (including the undefined “white” color for particles of non-homogeneous coloring). Statistical significance of the differences as computed by Tukey’s ‘Honest Significant Difference’ test (*: p < 0.01, ***: p < 0.001, ns: non-significant). (b) Schematic representation of the structural properties of the five colors for the *Drosophila* chromatin.

Overall, the chromatin colors resulted in distinct structural properties (Fig 5A). For example, black chromatin was the least accessible (median accessibility 26.5%), compared to green and blue (median accessibilities 34.4% and 34.3%, respectively) and to yellow and red (median accessibilities 46.5% and 51.6%, respectively). Black chromatin also featured the highest density in our models (median 212 bp/nm). This was slightly more than blue (207 bp/nm) and substantially more than green, yellow and red (182 bp/nm, 180 bp/nm, and 179 bp/nm, respectively). The chromatin type with most interactions was green (median 48.7 interacting particles within 250 nm) followed by black (45.3), yellow (43.7), blue (41.9), and red (37.9) chromatin. Finally, yellow and red chromatin featured the most extended fibers (median absolute angles 94.6° and 89.7°, respectively), compared to blue (85.3°), green (82.6°) and black (80.3°). Taken together, the 3D models generated by TADbit indicate that the chromatin types of *Drosophila* have intrinsic and distinctive structural properties.

It has been shown that the five types of *Drosophila* chromatin not only differ in protein composition but also in biochemical properties, transcriptional activity, histone modifications, replication timing, and DNA binding factors targeting [25]. They also differ in the sequence properties and the functions of the embedded genes. Now we demonstrate that the chromatin types also have specific and distinctive structural features (Fig 5B). Importantly, these results shed light on the nature of the elusive black chromatin. Most chromatin markers are depleted in this environment, including those responsible for active repression of transcription. It is thus unclear how genes are maintained silent and why transcription factors do not bind to their consensus sequence in black chromatin. Our results suggest that part of the answer is that black chromatin is very compact and inaccessible to external factors. The high curvature of black chromatin fibers in the models suggests that those regions are intrinsically ordered or that they are compressed. The enrichment of the linker histone H1 in black chromatin may account for all these properties. The previous conception of heterochromatin was closer to green (HP1-bound) or blue (Polycomb-bound) chromatin types. Interestingly, both of them are more accessible than black chromatin, yet green chromatin has a higher number of interactions. This indicates that green chromatin, compared to black chromatin, is a more open but irregular structure where specific interactions are more plausible within a distance cut-off. In contrast, the closed and regular organization of black chromatin results in fewer likely unspecific interactions per particle. This may somehow be related to the observation that the expression of some genes translocated to HP1-bound regions tends to fluctuate, a phenomenon known as position effect variegation [43]. We speculate that genes caught in this chromatin environment may be trapped in the local entanglement and physically locked away from their enhancers. Both yellow and red chromatin exhibit the most different structural features compared with black chromatin. Their 3D models are open and accessible, which is consistent with the fact that those regions are mostly transcribed and bound by many transcription factors. However, the overall protein occupancy in red chromatin is substantially higher than in yellow chromatin, yet their overall structural properties are relatively similar. This suggests that the extraordinary occupancy observed in red chromatin is not necessarily rooted in its conformational properties, but rather in mechanisms that operate at a finer scale. Additional studies will be needed to further investigate the molecular mechanisms associated to the structural properties of the chromatin types. However, our 3D models, as well as their correlation with the epigenetic features, are a firm basis for future investigation on chromatin occupancy by proteins and it spatial organization.

## Availability and future directions

Here we introduced TADbit to comprehensively address all the necessary steps from FASTQ files to the full analysis of 3D models. Currently, TADbit is the only computational pipeline to cover all relevant steps [44], including: (i) read quality control and design of the mapping strategy; (ii) mapping of reads to the reference genome; (iii) interaction map filtering and normalization; (iv) interaction matrix analysis, including matrix comparison, TAD detection and TAD alignment; (v) 3D modelling of genomes and genomic domains; and (vi) 3D model analysis. Recently, several publications have emerged comparing available tools, including TADbit, for a partial list of these steps. For example, reads mapping and contacts filtering [44–46] or TAD detection [34, 44]. Unfortunately, 3D modelling assessment is practically impossible given the lack of a golden set of genomes of known structure. However, initial theoretical assessments with toy models are being produced [21]. A complete list of the computational functions implemented in TADbit is provided in the Supplementary Material (S1 Text), each of which is more deeply described in the TADbit online documentation (http://3dgenomes.github.io/TADbit) together with complete tutorials covering each step from sequencing data to 3D model analysis. Next, we will further expand the TADbit functionality with additional modules for meta-matrix analysis, loop detection, matrix comparison, and additional features needed to fully analyze 3C based datasets.

## Supporting information

S1 Fig FASTQ quality control plots generated using the quality_plot function in TADbit. (a) Quality plots for the BR dataset. Top plot shows the PHRED score (blue line) and number of “N” positions (black line) as a function of the sequence position in the reads. Bottom plot shows the number of undigested sites (red), dangling ends (yellow) and re-ligated sites (blue) as a function of the nucleotide position in the reads. (b) TR1 dataset. (c) TR2 dataset. (d) SUM dataset. Panels b, c, and d show the same data as described in panel a.
(PDF)

S2 Fig Schematic representation of all applied filters in TADbit to remove 3C-based artifacts in the mapped reads. The filters include dangling-ends, self-circles, errors, random breaks, too short, too large, over-represented or duplicated reads. The exact definition of each of the filters can be found in the “online methods” section of the manuscript.
(PDF)

S3 Fig Percentage of borders of a given robustness score. Data for borders aligning within 10 kb (a) or exactly in the same bin (b). The plot on the left of the panels assesses the global sensitivity of TADbit predictions by comparing it with TAD borders “original definition” (see main text). The plot on the right assesses the sensitivity of TADbit prediction to experimental replicates. The plots show the border agreements (in percentage) as a function of the TADbit border strength.
(PDF)

S4 Fig 3D models of selected domains in the *Drosophila* genome. Superimposed 3D structures for selected models in cluster #1 for each of the 50 modeled domains. Models are colored by their particle chromatin type as previously defined [25]. They can be directly visualized using TADkit by visiting the Web site http://www.3DGenomes.org/datasets/serra_etal.
(PDF)

S1 Table Selected 50 regions of the *Drosophila melanogaster* genome for modeling. Columns in the table correspond to: Chromosome, starting coordinate, end coordinate, size of the region (in Mb), index number of the first bin (10Kb bins), index number of the last bin, size of the region (in bins), fraction of the different colors (white, red, yellow, green, blue and black), MMP score [21] of the interaction matrix, correlation coefficient of the contact map of the final models and the initial interaction matrix, TADbit parameters for the modeling (including, scale, lower distance, lower cut-off, upper cut-off, and distance cut-off). Description of the parameters can be found in the main text.
(PDF)

S1 Text TADbit classes and functions. Description of all functions and classes available in TADbit.
(PDF)

S2 Text TAD border detection from interaction matrices. Outline of the TAD detection algorithm implemented in TADbit.
(PDF)

S3 Text Structural similarity score for model clustering. Description of the similarity measure comparing two models generated with TADbit.
(PDF)

S4 Text Serra et al 2015 dataset. Description of the Serra et al 2015 dataset to reproduce the data in this article.
(PDF)

## References

[1] Cremer T, Cremer C. Chromosome territories, nuclear architecture and gene regulation in mammalian cells. Nat Rev Genet. 2001;2(4):292–301. doi: 10.1038/35066075 11283701 10.1038/35066075

[2] Lieberman-Aiden E, van Berkum NL, Williams L, Imakaev M, Ragoczy T, Telling A, et al Comprehensive mapping of long-range interactions reveals folding principles of the human genome. Science. 2009;326(5950):289–93. doi: 10.1126/science.1181369 19815776 10.1126/science.1181369 PMC2858594

[3] Dixon JR, Selvaraj S, Yue F, Kim A, Li Y, Shen Y, et al Topological domains in mammalian genomes identified by analysis of chromatin interactions. Nature. 2012;485(7398):376–80. doi: 10.1038/nature11082 22495300 10.1038/nature11082 PMC3356448

[4] Nora EP, Lajoie BR, Schulz EG, Giorgetti L, Okamoto I, Servant N, et al Spatial partitioning of the regulatory landscape of the X-inactivation centre. Nature. 2012;485(7398):381–5. doi: 10.1038/nature11049 22495304 10.1038/nature11049 PMC3555144

[5] Sexton T, Yaffe E, Kenigsberg E, Bantignies F, Leblanc B, Hoichman M, et al Three-dimensional folding and functional organization principles of the Drosophila genome. Cell. 2012;148(3):458–72. doi: 10.1016/j.cell.2012.01.010 22265598 10.1016/j.cell.2012.01.010

[6] Takizawa T, Meaburn KJ, Misteli T. The meaning of gene positioning. Cell. 2008;135(1):9–13. doi: 10.1016/j.cell.2008.09.026 18854147 10.1016/j.cell.2008.09.026 PMC3478881

[7] Dekker J, Rippe K, Dekker M, Kleckner N. Capturing chromosome conformation. Science. 2002;295(5558):1306–11. doi: 10.1126/science.1067799 11847345 10.1126/science.1067799

[8] Dekker J, Marti-Renom MA, Mirny LA. Exploring the three-dimensional organization of genomes: interpreting chromatin interaction data. Nat Rev Genet. 2013;14(6):390–403. doi: 10.1038/nrg3454 23657480 10.1038/nrg3454 PMC3874835

[9] Serra F, Di Stefano M, Spill YG, Cuartero Y, Goodstadt M, Bau D, et al Restraint-based three-dimensional modeling of genomes and genomic domains. FEBS Lett. 2015;589(20 Pt A):2987–95. doi: 10.1016/j.febslet.2015.05.012 25980604 10.1016/j.febslet.2015.05.012

[10] Zhang Z, Li G, Toh KC, Sung WK. 3D chromosome modeling with semi-definite programming and Hi-C data. J Comput Biol. 2013;20(11):831–46. doi: 10.1089/cmb.2013.0076 24195706 10.1089/cmb.2013.0076

[11] Lesne A, Riposo J, Roger P, Cournac A, Mozziconacci J. 3D genome reconstruction from chromosomal contacts. Nat Methods. 2014;11(11):1141–3. doi: 10.1038/nmeth.3104 25240436 10.1038/nmeth.3104

[12] Baù D, Marti-Renom MA. Genome structure determination via 3C-based data integration by the Integrative Modeling Platform. Methods. 2012;58(3):300–6. doi: 10.1016/j.ymeth.2012.04.004 22522224 10.1016/j.ymeth.2012.04.004

[13] Hu M, Deng K, Qin Z, Dixon J, Selvaraj S, Fang J, et al Bayesian inference of spatial organizations of chromosomes. PLoS Comput Biol. 2013;9(1):e1002893 doi: 10.1371/journal.pcbi.1002893 23382666 10.1371/journal.pcbi.1002893 PMC3561073

[14] Giorgetti L, Galupa R, Nora EP, Piolot T, Lam F, Dekker J, et al Predictive polymer modeling reveals coupled fluctuations in chromosome conformation and transcription. Cell. 2014;157(4):950–63. Epub 2014/05/13. doi: 10.1016/j.cell.2014.03.025 24813616 10.1016/j.cell.2014.03.025 PMC4427251

[15] Duan Z, Andronescu M, Schutz K, McIlwain S, Kim YJ, Lee C, et al A three-dimensional model of the yeast genome. Nature. 2010;465(7296):363 doi: 10.1038/nature08973 20436457 10.1038/nature08973 PMC2874121

[16] Rousseau M, Fraser J, Ferraiuolo MA, Dostie J, Blanchette M. Three-dimensional modeling of chromatin structure from interaction frequency data using Markov chain Monte Carlo sampling. BMC Bioinformatics. 2011;12:414 doi: 10.1186/1471-2105-12-414 22026390 10.1186/1471-2105-12-414 PMC3245522

[17] Varoquaux N, Ay F, Noble WS, Vert JP. A statistical approach for inferring the 3D structure of the genome. Bioinformatics. 2014;30(12):i26–33. doi: 10.1093/bioinformatics/btu268 24931992 10.1093/bioinformatics/btu268 PMC4229903

[18] Meluzzi D, Arya G. Recovering ensembles of chromatin conformations from contact probabilities. Nucleic Acids Res. 2013;41(1):63–75. doi: 10.1093/nar/gks1029 23143266 10.1093/nar/gks1029 PMC3592477

[19] Peng C, Fu LY, Dong PF, Deng ZL, Li JX, Wang XT, et al The sequencing bias relaxed characteristics of Hi-C derived data and implications for chromatin 3D modeling. Nucleic Acids Res. 2013;41(19):e183 doi: 10.1093/nar/gkt745 23965308 10.1093/nar/gkt745 PMC3799458

[20] Kalhor R, Tjong H, Jayathilaka N, Alber F, Chen L. Genome architectures revealed by tethered chromosome conformation capture and population-based modeling. Nat Biotechnol. 2011;30(1):90–8. doi: 10.1038/nbt.2057 22198700 10.1038/nbt.2057 PMC3782096

[21] Trussart M, Serra F, Bau D, Junier I, Serrano L, Marti-Renom MA. Assessing the limits of restraint-based 3D modeling of genomes and genomic domains. Nucleic Acids Res. 2015;43(7):3465–77. doi: 10.1093/nar/gkv221 25800747 10.1093/nar/gkv221 PMC4402535

[22] Le Dily F, Bau D, Pohl A, Vicent GP, Serra F, Soronellas D, et al Distinct structural transitions of chromatin topological domains correlate with coordinated hormone-induced gene regulation. Genes Dev. 2014;28(19):2151–62. doi: 10.1101/gad.241422.114 25274727 10.1101/gad.241422.114 PMC4180976

[23] Umbarger MA, Toro E, Wright MA, Porreca GJ, Bau D, Hong SH, et al The three-dimensional architecture of a bacterial genome and its alteration by genetic perturbation. Mol Cell. 2011;44(2):252–64. doi: 10.1016/j.molcel.2011.09.010 22017872 10.1016/j.molcel.2011.09.010 PMC3874842

[24] Baù D, Sanyal A, Lajoie BR, Capriotti E, Byron M, Lawrence JB, et al The three-dimensional folding of the alpha-globin gene domain reveals formation of chromatin globules. Nat Struct Mol Biol. 2011;18(1):107–14. doi: 10.1038/nsmb.1936 21131981 10.1038/nsmb.1936 PMC3056208

[25] Filion GJ, van Bemmel JG, Braunschweig U, Talhout W, Kind J, Ward LD, et al Systematic protein location mapping reveals five principal chromatin types in Drosophila cells. Cell. 2010;143(2):212–24. doi: 10.1016/j.cell.2010.09.009 20888037 10.1016/j.cell.2010.09.009 PMC3119929

[26] Hou C, Li L, Qin ZS, Corces VG. Gene density, transcription, and insulators contribute to the partition of the Drosophila genome into physical domains. Mol Cell. 2012;48(3):471–84. doi: 10.1016/j.molcel.2012.08.031 23041285 10.1016/j.molcel.2012.08.031 PMC3496039

[27] Le Dily F, Baù D, Pohl A, Vicent GP, Serra F, Soronellas D, et al Distinct structural transitions of chromatin topological domains correlate with coordinated hormone-induced gene regulation. Genes Dev. 2014;28(19):2151–62. doi: 10.1101/gad.241422.114 25274727 10.1101/gad.241422.114 PMC4180976

[28] Rao SS, Huntley MH, Durand NC, Stamenova EK, Bochkov ID, Robinson JT, et al A 3D Map of the Human Genome at Kilobase Resolution Reveals Principles of Chromatin Looping. Cell. 2014;159(7):1665–80. doi: 10.1016/j.cell.2014.11.021 25497547 10.1016/j.cell.2014.11.021 PMC5635824

[29] Mizuguchi T, Fudenberg G, Mehta S, Belton JM, Taneja N, Folco HD, et al Cohesin-dependent globules and heterochromatin shape 3D genome architecture in S. pombe. Nature. 2014;516(7531):432–5. doi: 10.1038/nature13833 25307058 10.1038/nature13833 PMC4465753

[30] Phillips-Cremins JE, Sauria ME, Sanyal A, Gerasimova TI, Lajoie BR, Bell JS, et al Architectural protein subclasses shape 3D organization of genomes during lineage commitment. Cell. 2013;153(6):1281–95. Epub 2013/05/28. doi: 10.1016/j.cell.2013.04.053 PubMed Central PMCID: PMC3712340. 23706625 10.1016/j.cell.2013.04.053 PMC3712340

[31] Andrews S. FastQC: A quality control tool for high throughput sequence data. 2010. Available from: http://www.bioinformatics.babraham.ac.uk/projects/fastqc/.

[32] Imakaev M, Fudenberg G, McCord RP, Naumova N, Goloborodko A, Lajoie BR, et al Iterative correction of Hi-C data reveals hallmarks of chromosome organization. Nat Methods. 2012;9(10):999–1003. doi: 10.1038/nmeth.2148 22941365 10.1038/nmeth.2148 PMC3816492

[33] Marco-Sola S, Sammeth M, Guigo R, Ribeca P. The GEM mapper: fast, accurate and versatile alignment by filtration. Nat Methods. 2012;9(12):1185–8. doi: 10.1038/nmeth.2221 23103880 10.1038/nmeth.2221

[34] Dali R, Blanchette M. A critical assessment of topologically associating domain prediction tools. Nucleic Acids Res. 2017;45(6):2994–3005. doi: 10.1093/nar/gkx145 28334773 10.1093/nar/gkx145 PMC5389712

[35] Needleman SB, Wunsch CD. A general method applicable to the search for similarities in the amino acid sequence of two proteins. J Mol Biol. 1970;48:443–53. 5420325 10.1016/0022-2836(70)90057-4

[36] Russel D, Lasker K, Webb B, Velazquez-Muriel J, Tjioe E, Schneidman-Duhovny D, et al Putting the pieces together: integrative modeling platform software for structure determination of macromolecular assemblies. PLoS Biol. 2012;10(1):e1001244 doi: 10.1371/journal.pbio.1001244 22272186 10.1371/journal.pbio.1001244 PMC3260315

[37] Bau D, Martin AJ, Mooney C, Vullo A, Walsh I, Pollastri G. Distill: a suite of web servers for the prediction of one-, two- and three-dimensional structural features of proteins. BMC Bioinformatics. 2006;7:402 doi: 10.1186/1471-2105-7-402 16953874 10.1186/1471-2105-7-402 PMC1574355

[38] Enright AJ, Van Dongen S, Ouzounis CA. An efficient algorithm for large-scale detection of protein families. Nucleic Acids Res. 2002;30(7):1575–84. 11917018 10.1093/nar/30.7.1575 PMC101833

[39] SRA toolkit. Available from: http://www.ncbi.nlm.nih.gov/Traces/sra/sra.cgi?cmd=show&f=software&m=software&s=software.

[40] Ewing B, Hillier L, Wendl MC, Green P. Base-calling of automated sequencer traces using phred. I. Accuracy assessment. Genome Res. 1998;8(3):175–85. 9521921 10.1101/gr.8.3.175

[41] Olshen AB, Venkatraman ES, Lucito R, Wigler M. Circular binary segmentation for the analysis of array-based DNA copy number data. Biostatistics. 2004;5(4):557–72. Epub 2004/10/12. doi: 10.1093/biostatistics/kxh008 15475419 10.1093/biostatistics/kxh008

[42] Gerchman SE, Ramakrishnan V. Chromatin higher-order structure studied by neutron scattering and scanning transmission electron microscopy. Proc Natl Acad Sci U S A. 1987;84(22):7802–6. 3479765 10.1073/pnas.84.22.7802 PMC299397

[43] Elgin SC, Reuter G. Position-effect variegation, heterochromatin formation, and gene silencing in Drosophila. Cold Spring Harb Perspect Biol. 2013;5(8):a017780 Epub 2013/08/03. doi: 10.1101/cshperspect.a017780 23906716 10.1101/cshperspect.a017780 PMC3721279

[44] Shavit Y, Merelli I, Milanesi L, Lio P. How computer science can help in understanding the 3D genome architecture. Brief Bioinform. 2016;17(5):733–44. doi: 10.1093/bib/bbv085 26433013 10.1093/bib/bbv085

[45] Lazaris C, Kelly S, Ntziachristos P, Aifantis I, Tsirigos A. HiC-bench: comprehensive and reproducible Hi-C data analysis designed for parameter exploration and benchmarking. BMC Genomics. 2017;18(1):22 doi: 10.1186/s12864-016-3387-6 28056762 10.1186/s12864-016-3387-6 PMC5217551

[46] Ay F, Noble WS. Analysis methods for studying the 3D architecture of the genome. Genome Biol. 2015;16:183 doi: 10.1186/s13059-015-0745-7 26328929 10.1186/s13059-015-0745-7 PMC4556012

[47] Pettersen EF, Goddard TD, Huang CC, Couch GS, Greenblatt DM, Meng EC, et al UCSF Chimera—a visualization system for exploratory research and analysis. J Comput Chem. 2004;25(13):1605–12. doi: 10.1002/jcc.20084 15264254 10.1002/jcc.20084

