# Supplementary material for: Automatic analysis and 3D-modelling of Hi-C data using TADbit reveals structural features of the fly chromatin colors
Source: PLoS Comput Biol. 2017 Jul 19;13(7):e1005665. doi: 10.1371/journal.pcbi.1005665 (PMC5540598; doi:10.1371/journal.pcbi.1005665)

Figure S1. FASTQ quality control plots generated using the quality_plot function in TADbit. (a) Quality plots for the BR dataset. Top plot shows the PHRED score (blue line) and number of “N” positions (black line) as a function of the sequence position in the reads. Bottom plot shows the number of undigested sites (red), dangling ends (yellow) and re-ligated sites (blue) as a function of the nucleotide position in the reads. (b) TR1 dataset. (c) TR2 dataset. (d) SUM dataset. Panels b, c, and d show the same data as described in panel a

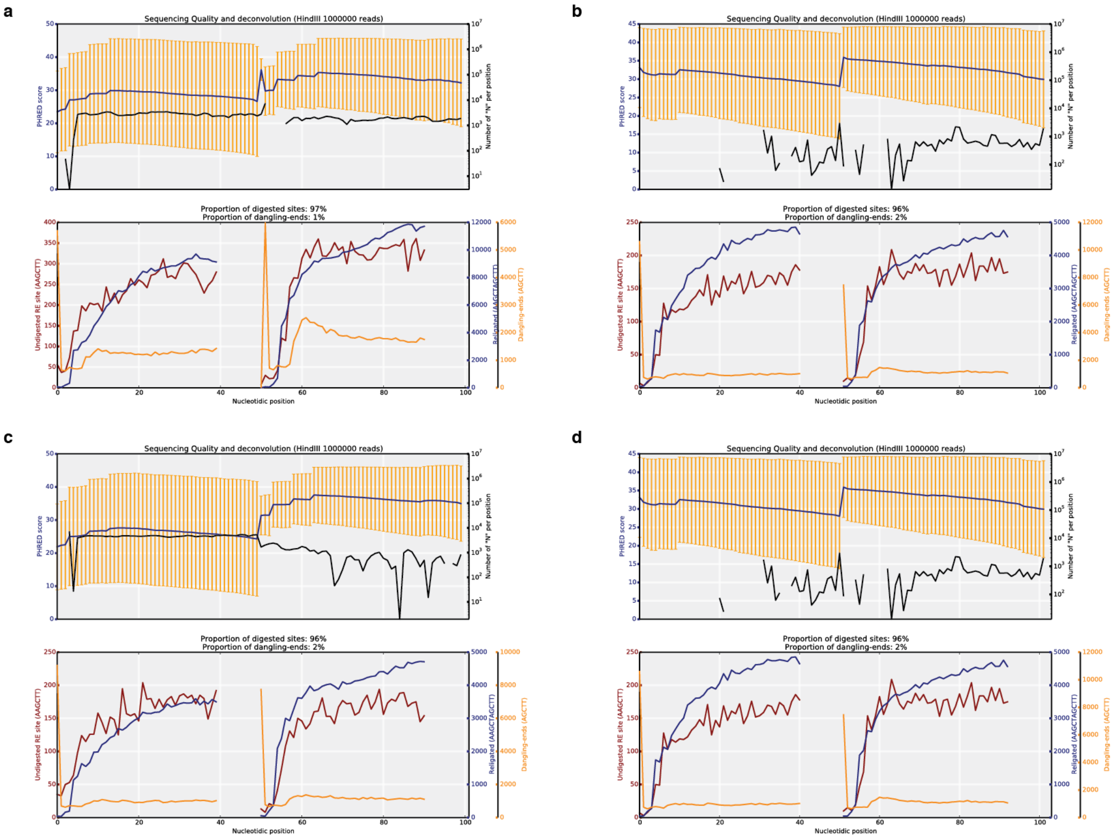

Supplement: S1 Fig — (a) Quality plots for the BR dataset. Top plot shows the PHRED score (blue line) and number of “N” positions (black line) as a function of the sequence position in the reads. Bottom plot shows the number of undigested sites (red), dangling ends (yellow) and re-ligated sites (blue) as a function of the nucleotide position in the reads. (b) TR1 dataset. (c) TR2 dataset. (d) SUM dataset. Panels b, c, and d show the same data as described in panel a. (PDF) [file pcbi.1005665.s001.pdf]
