# Supplementary material for: Automatic analysis and 3D-modelling of Hi-C data using TADbit reveals structural features of the fly chromatin colors
Source: PLoS Comput Biol. 2017 Jul 19;13(7):e1005665. doi: 10.1371/journal.pcbi.1005665 (PMC5540598; doi:10.1371/journal.pcbi.1005665)

**Figure S2. Schematic representation of all applied filters in TADbit to remove 3C-based artifacts in the mapped reads**. The filters include dangling-ends, self-circles, errors, random breaks, too short, too large, over-represented or duplicated reads. The exact definition of each of the filters can be found in the "online methods" section of the manuscript.

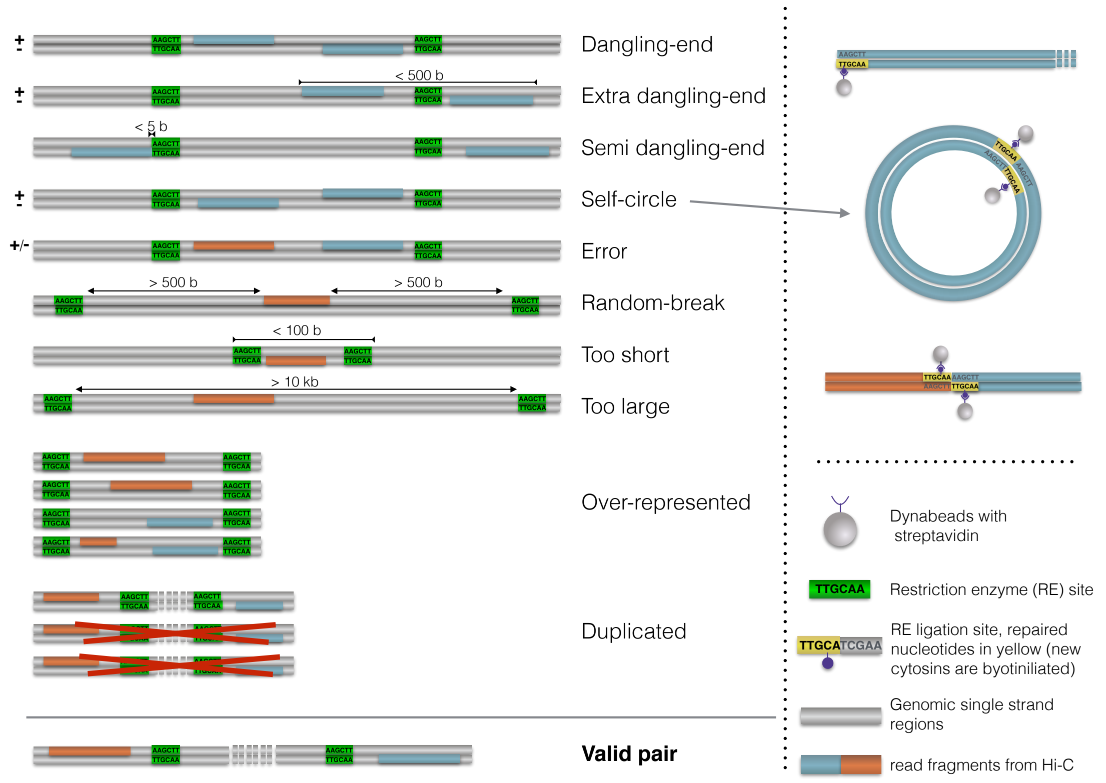

Supplement: S2 Fig — The filters include dangling-ends, self-circles, errors, random breaks, too short, too large, over-represented or duplicated reads. The exact definition of each of the filters can be found in the “online methods” section of the manuscript. (PDF) [file pcbi.1005665.s002.pdf]
