# Supplementary material for: Automatic analysis and 3D-modelling of Hi-C data using TADbit reveals structural features of the fly chromatin colors
Source: PLoS Comput Biol. 2017 Jul 19;13(7):e1005665. doi: 10.1371/journal.pcbi.1005665 (PMC5540598; doi:10.1371/journal.pcbi.1005665)

Figure S3. Percentage of borders of a given robustness score. Data for borders aligning within 10 kb (a) or exactly in the same bin (b). The plot on the left of the panels assesses the global sensitivity of TADbit predictions by comparing it with TAD borders “original definition” (see main text). The plot on the right assesses the sensitivity of TADbit prediction to experimental replicates. The plots show the border agreements (in percentage) as a function of the TADbit border strength.

a

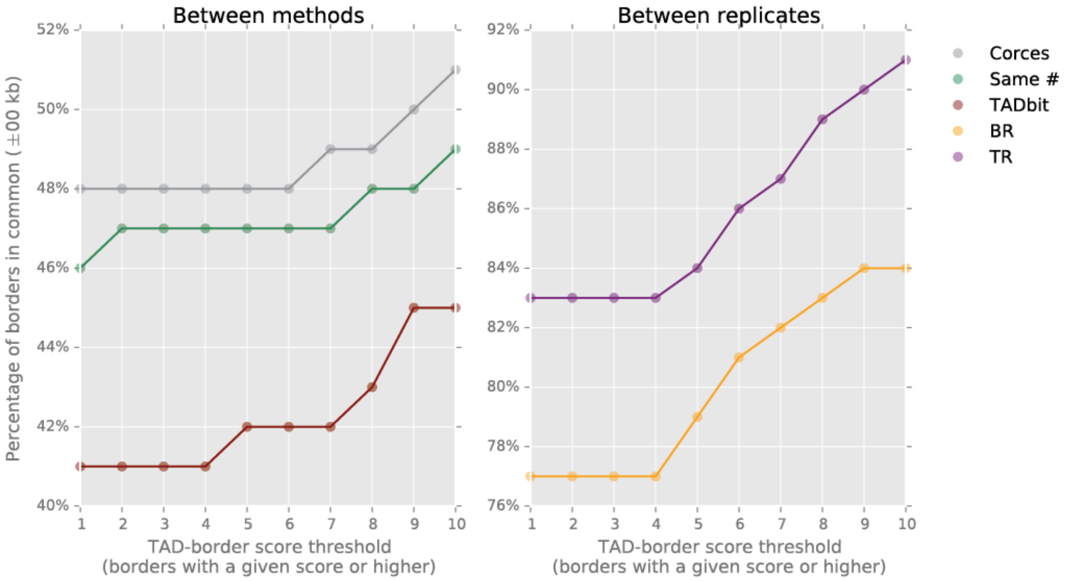

b

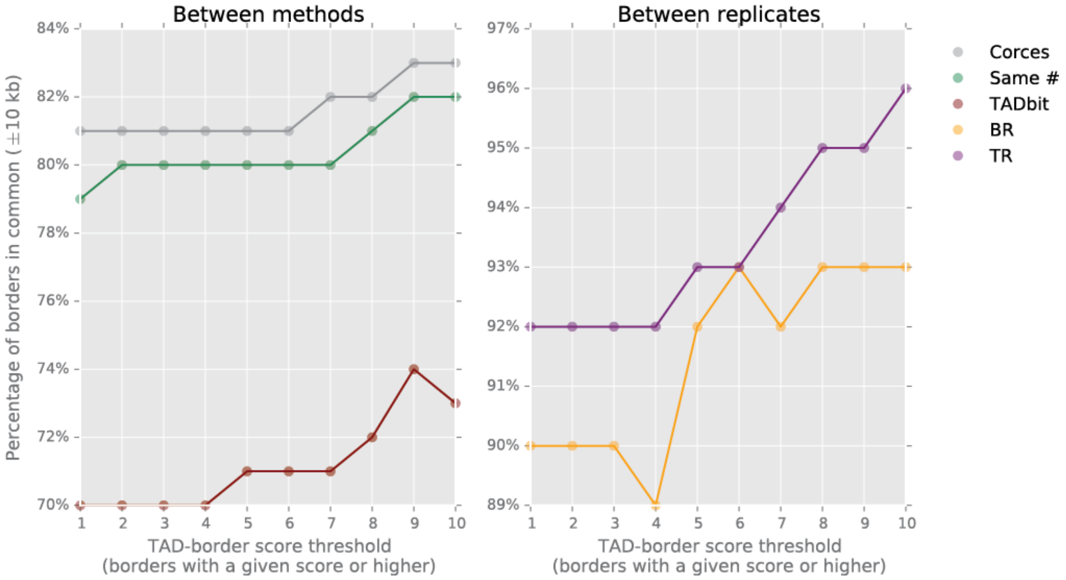

Supplement: S3 Fig — Data for borders aligning within 10 kb (a) or exactly in the same bin (b). The plot on the left of the panels assesses the global sensitivity of TADbit predictions by comparing it with TAD borders “original definition” (see main text). The plot on the right assesses the sensitivity of TADbit prediction to experimental replicates. The plots show the border agreements (in percentage) as a function of the TADbit border strength. (PDF) [file pcbi.1005665.s003.pdf]
