# Supplementary material for: Automatic analysis and 3D-modelling of Hi-C data using TADbit reveals structural features of the fly chromatin colors
Source: PLoS Comput Biol. 2017 Jul 19;13(7):e1005665. doi: 10.1371/journal.pcbi.1005665 (PMC5540598; doi:10.1371/journal.pcbi.1005665)

Figure S4. 3D models of selected domains in the *Drosophila* genome. Superimposed 3D structures for selected models in cluster #1 for each of the 50 modeled domains. Models are colored by their particle chromatin type as previously defined [1]. They can be directly visualized using TADkit by visiting the Web site http://www.3DGenomes.org/datasets/serra_etal.

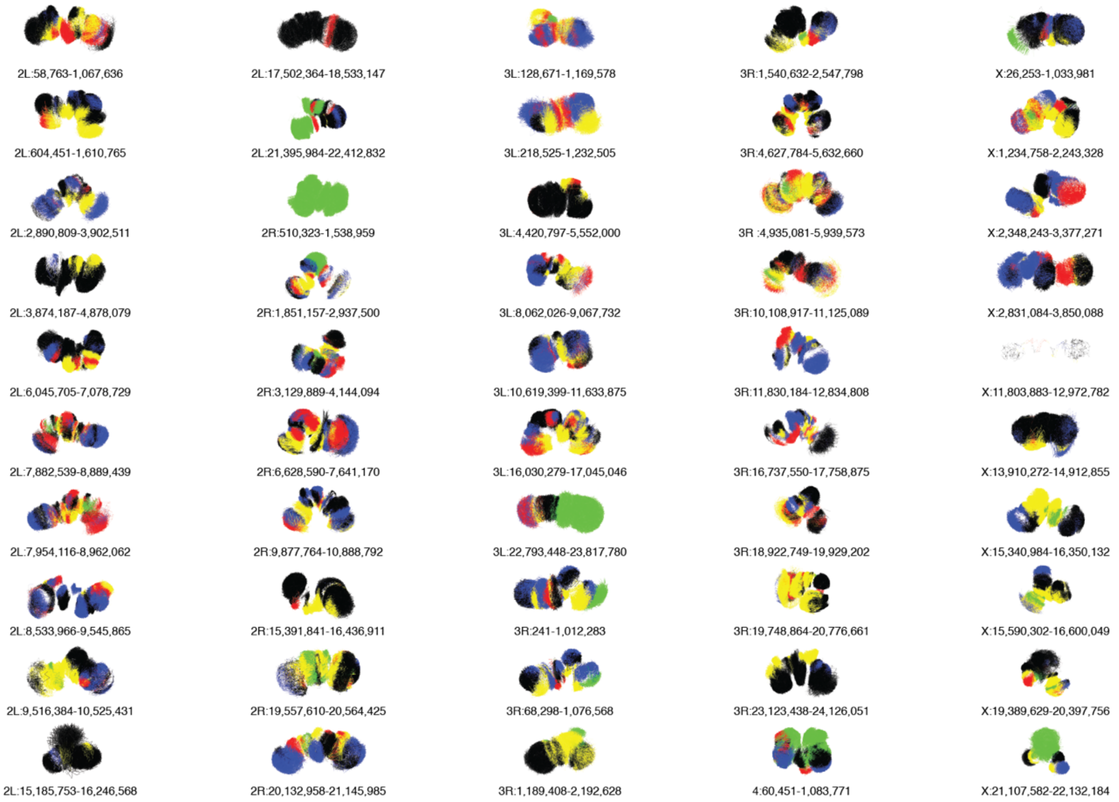

Supplement: S4 Fig — Superimposed 3D structures for selected models in cluster #1 for each of the 50 modeled domains. Models are colored by their particle chromatin type as previously defined [25]. They can be directly visualized using TADkit by visiting the Web site http://www.3DGenomes.org/datasets/serra_etal. (PDF) [file pcbi.1005665.s004.pdf]
