# Supplementary material for: Automatic analysis and 3D-modelling of Hi-C data using TADbit reveals structural features of the fly chromatin colors
Source: PLoS Comput Biol. 2017 Jul 19;13(7):e1005665. doi: 10.1371/journal.pcbi.1005665 (PMC5540598; doi:10.1371/journal.pcbi.1005665)
Supplement: S1 Table — Columns in the table correspond to: Chromosome, starting coordinate, end coordinate, size of the region (in Mb), index number of the first bin (10Kb bins), index number of the last bin, size of the region (in bins), fraction of the different colors (white, red, yellow, green, blue and black), MMP score [21] of the interaction matrix, correlation coefficient of the contact map of the final models and the initial interaction matrix, TADbit parameters for the modeling (including, scale, lower distance, lower cut-off, upper cut-off, and distance cut-off). Description of the parameters can be found in the main text. (PDF) [file pcbi.1005665.s005.pdf]

Table S1. Selected 50 regions of the *Drosophila melanogaster* genome for modeling. Columns in the table correspond to: Chromosome, starting coordinate, end coordinate, size of the region (in Mb), index number of the first bin (10Kb bins), index number of the last bin, size of the region (in bins), fraction of the different colors (white, red, yellow, green, blue and black), MMP score [2] of the interaction matrix, correlation coefficient of the contact map of the final models and the initial interaction matrix, TADbit parameters for the modeling (including, scale, lower distance, lower cut-off, upper cut-off, and distance cut-off). Description of the parameters can be found in the main text.

R#	Chr.	Start coordinate	End coordinate	Size (Mb)	Start bin	End bin	Bin size	Fraction white	Fraction red	Fraction yellow	Fraction green	Fraction blue	Fraction black	MMP Score	Corre. Hi-C	Scale	Lower dist	Lower cutoff	Upper cutoff	Dist. cutoff
1	chr2L	58,763	1,067,636	1.01	5	106	102	0.04	0.16	0.28	0.00	0.05	0.47	0.93	0.90	0.01	400	0.0	-0.6	3
2	chr2L	604,451	1,610,765	1.01	60	161	102	0.01	0.05	0.13	0.00	0.11	0.71	0.95	0.91	0.01	400	-0.3	-0.9	3
3	chr2L	2,890,809	3,902,511	1.01	289	390	102	0.10	0.03	0.18	0.00	0.31	0.38	0.87	0.89	0.01	400	0.3	-0.9	3
4	chr2L	3,874,187	4,878,079	1.00	387	487	101	0.03	0.00	0.07	0.00	0.02	0.88	0.94	0.89	0.01	300	-0.3	-0.6	2
5	chr2L	6,045,705	7,078,729	1.03	604	707	104	0.04	0.01	0.29	0.01	0.12	0.54	0.90	0.89	0.01	400	0.0	-0.9	3
6	chr2L	7,882,539	8,889,439	1.01	788	888	101	0.07	0.16	0.18	0.08	0.26	0.26	0.91	0.88	0.01	400	0.0	-0.6	3
7	chr2L	7,954,116	8,962,062	1.01	795	896	102	0.09	0.16	0.19	0.08	0.24	0.24	0.91	0.90	0.01	400	0.0	-0.6	3
8	chr2L	8,533,966	9,545,865	1.01	853	954	102	0.08	0.07	0.04	0.00	0.35	0.46	0.89	0.91	0.01	400	0.0	-0.6	3
9	chr2L	9,516,384	10,525,431	1.01	951	1052	102	0.06	0.05	0.32	0.01	0.13	0.43	0.90	0.87	0.01	400	0.0	-1.2	3
10	chr2L	15,185,753	16,246,568	1.06	1518	1624	107	0.01	0.00	0.05	0.00	0.03	0.92	0.92	0.92	0.01	300	-0.3	-0.9	2
11	chr2L	17,502,364	18,533,147	1.03	1750	1853	104	0.01	0.01	0.00	0.00	0.00	0.98	0.88	0.92	0.01	200	0.0	0.0	2
12	chr2L	21,395,984	22,412,832	1.02	2139	2241	103	0.04	0.03	0.01	0.41	0.18	0.33	0.94	0.92	0.01	400	0.0	-0.9	3
13	chr2R	510,323	1,538,959	1.03	51	153	103	0.00	0.00	0.00	1.00	0.00	0.00	0.85	0.88	0.01	400	0.0	-0.9	3
14	chr2R	1,851,157	2,937,500	1.09	185	293	102	0.07	0.07	0.21	0.23	0.20	0.23	0.91	0.86	0.01	400	0.0	-0.6	3
15	chr2R	3,129,889	4,144,094	1.01	312	414	103	0.07	0.12	0.36	0.04	0.17	0.25	0.90	0.85	0.01	400	0.0	-0.6	3
16	chr2R	6,628,590	7,641,170	1.01	662	764	103	0.04	0.13	0.15	0.00	0.29	0.40	0.90	0.87	0.01	400	0.0	-0.6	3
17	chr2R	9,877,764	10,888,792	1.01	987	1088	102	0.08	0.10	0.21	0.00	0.30	0.31	0.92	0.87	0.01	400	0.0	-0.3	3
18	chr2R	15,391,841	16,436,911	1.05	1539	1643	102	0.01	0.02	0.07	0.00	0.01	0.89	0.94	0.90	0.01	300	-0.3	-0.6	2
19	chr2R	19,557,610	20,564,425	1.01	1955	2056	102	0.06	0.03	0.40	0.06	0.13	0.32	0.91	0.89	0.01	400	0.0	-0.6	3
20	chr2R	20,132,958	21,145,985	1.01	2013	2114	102	0.07	0.06	0.17	0.03	0.39	0.28	0.93	0.90	0.01	400	0.0	-0.9	3
21	chr3L	128,671	1,169,578	1.04	12	116	105	0.09	0.11	0.22	0.02	0.44	0.12	0.92	0.88	0.01	400	0.0	-0.9	3
22	chr3L	218,525	1,232,505	1.01	21	123	103	0.08	0.11	0.17	0.00	0.52	0.13	0.93	0.89	0.01	300	0.0	-0.6	2
23	chr3L	4,420,797	5,552,000	1.13	442	555	114	0.02	0.04	0.04	0.02	0.04	0.86	0.91	0.90	0.01	300	-0.3	-0.6	2
24	chr3L	8,062,026	9,067,732	1.01	806	906	101	0.09	0.09	0.25	0.00	0.21	0.37	0.87	0.87	0.01	400	0.0	-0.9	3
25	chr3L	10,619,399	11,633,875	1.01	1061	1163	103	0.08	0.15	0.17	0.00	0.44	0.17	0.93	0.85	0.01	300	0.0	-0.6	2
26	chr3L	16,030,279	17,045,046	1.01	1603	1704	102	0.04	0.09	0.31	0.00	0.16	0.40	0.86	0.85	0.01	400	0.0	-0.9	3
27	chr3L	22,793,448	23,817,780	1.02	2279	2381	103	0.01	0.06	0.04	0.73	0.04	0.13	0.88	0.93	0.01	200	0.0	-0.6	2
28	chr3R	20,000	1,012,283	0.99	2	101	100	0.08	0.03	0.15	0.08	0.47	0.20	0.91	0.89	0.01	400	0.0	-0.9	3
29	chr3R	68,298	1,076,568	1.01	6	107	102	0.09	0.03	0.17	0.03	0.46	0.23	0.91	0.89	0.01	400	0.0	-0.6	3
30	chr3R	1,189,408	2,192,628	1.00	118	219	102	0.02	0.00	0.22	0.07	0.00	0.70	0.92	0.91	0.01	300	-0.3	-0.9	2
31	chr3R	1,540,632	2,547,798	1.01	154	254	101	0.03	0.03	0.11	0.01	0.06	0.76	0.92	0.89	0.01	300	0.0	-0.9	2
32	chr3R	4,627,784	5,632,660	1.00	462	563	102	0.07	0.10	0.32	0.02	0.13	0.36	0.90	0.86	0.01	400	0.0	-0.6	3
33	chr3R	4,935,081	5,939,573	1.00	493	593	101	0.05	0.09	0.30	0.02	0.18	0.37	0.90	0.88	0.01	400	0.3	-0.9	3
34	chr3R	10,108,917	11,125,089	1.02	1010	1112	103	0.06	0.15	0.15	0.01	0.15	0.47	0.91	0.90	0.01	400	0.3	-0.9	3
35	chr3R	11,830,184	12,834,808	1.00	1183	1283	101	0.06	0.08	0.11	0.00	0.49	0.26	0.95	0.89	0.01	400	-0.3	-0.6	3
36	chr3R	16,737,550	17,758,875	1.02	1673	1775	103	0.13	0.14	0.13	0.00	0.27	0.34	0.87	0.85	0.01	400	0.0	0.0	3
37	chr3R	18,922,749	19,929,202	1.01	1892	1992	101	0.06	0.22	0.17	0.00	0.11	0.45	0.92	0.85	0.01	400	-0.3	-0.6	3
38	chr3R	19,748,864	20,776,661	1.03	1974	2077	104	0.05	0.14	0.39	0.04	0.02	0.38	0.92	0.83	0.01	400	-0.3	-0.6	3
39	chr3R	23,123,438	24,126,051	1.00	2312	2412	101	0.01	0.00	0.13	0.00	0.05	0.81	0.94	0.89	0.01	300	-0.3	-0.6	2
40	chr4	60,451	1,083,771	1.02	6	108	102	0.03	0.06	0.01	0.51	0.20	0.20	0.87	0.88	0.01	400	0.0	-1.2	3
41	chrX	26,253	1,033,981	1.01	2	103	102	0.05	0.05	0.15	0.01	0.31	0.43	0.95	0.89	0.01	300	-0.3	-0.6	2
42	chrX	1,234,758	2,243,328	1.01	123	224	102	0.07	0.11	0.31	0.05	0.15	0.31	0.88	0.85	0.01	400	0.0	-0.9	3
43	chrX	2,348,243	3,377,271	1.03	234	337	104	0.04	0.11	0.12	0.01	0.48	0.25	0.94	0.89	0.01	400	0.0	-0.6	3
44	chrX	2,831,084	3,850,088	1.02	283	385	103	0.06	0.17	0.06	0.00	0.44	0.27	0.95	0.89	0.01	400	0.3	-0.6	3
45	chrX	11,803,883	12,972,782	1.17	1180	1297	118	0.04	0.05	0.08	0.00	0.07	0.76	0.92	0.89	0.01	400	0.3	-0.9	3
46	chrX	13,910,272	14,912,855	1.00	1391	1491	101	0.02	0.02	0.05	0.00	0.08	0.83	0.93	0.90	0.01	300	-0.3	-1.2	2
47	chrX	15,340,984	16,350,132	1.01	1534	1635	102	0.06	0.01	0.42	0.06	0.24	0.21	0.92	0.89	0.01	400	0.0	-0.6	3
48	chrX	15,590,302	16,600,049	1.01	1559	1660	102	0.03	0.00	0.48	0.04	0.20	0.26	0.92	0.87	0.01	400	0.0	-0.9	3
49	chrX	19,389,629	20,397,756	1.01	1938	2039	102	0.09	0.04	0.23	0.09	0.06	0.50	0.90	0.89	0.01	400	-0.3	-0.3	3
50	chrX	21,107,582	22,132,184	1.02	2110	2213	103	0.06	0.00	0.11	0.37	0.11	0.36	0.96	0.88	0.01	400	0.0	-0.6	3
