# Supplementary material for: Automatic analysis and 3D-modelling of Hi-C data using TADbit reveals structural features of the fly chromatin colors
Source: PLoS Comput Biol. 2017 Jul 19;13(7):e1005665. doi: 10.1371/journal.pcbi.1005665 (PMC5540598; doi:10.1371/journal.pcbi.1005665)
Supplement: S1 Text — Description of all functions and classes available in TADbit. (PDF) [file pcbi.1005665.s006.pdf]

S1 Text. TADbit classes and functions
Principal function, with their (non-default) parameters, used for the analysis of the fly genome (variables starting with a *$* are path to input/output files or folders).

- **quality_plot** *($FASTQ, nreads=1000000, r_enz='HindIII', paired=True)*
- **full_mapping** *($INDEX, $FASTQ, $OUTMAPPED, windows=((5, 20), (5, 30), (5, 40), (5, 50), (10, 30), (10, 40), (10, 50), (20, 40), (20, 50)), frag_map=False, r_enz='HindIII')*
- **parse_map** *($OUTMAPPED_read1, $OUTMAPPED_read2, $PARSED_read1, $PARSED_read2, re_name='HindIII')*
- **get_intersection** *($PARSED_read1, $PARSED_read2, $INTERSECTION, verbose=True)*
- **plot_distance_vs_interactions** *($INTERSECTION, max_diff=50000000, resolution-10000)*
- **plot_genomic_distribution** *($INTERSECTION, resolution=1000000)*
- **hic_map** *($INTERSECTION, resolution=100000, cmap="tadbit")*
- **filter_reads** *($INTERSECTION, max_molecule_length=500, over_represented=0.001, max_frag_size=100000, min_frag_size=50, re_proximity=5)*
- **apply_filters** *($INTERSECTION, $FILTERED, filters=None)*
- **filter_columns** *(draw_hist=True)*
- **normalize_hic** *(max_dev=0.1, iterations=10)*
- **correlate_matrices** *($GENOMIC_MATRIX1, $GENOMIC_MATRIX2, max_dist=200)*
- **eig_correlate_matrices** *($GENOMIC_MATRIX1, $GENOMIC_MATRIX2, nvect=10)*
- **find_tad** *(n_cpus=20, verbose=False)*
- **align_experiments** *(randomize=True, rnd_method="interpolate", rnd_num=1000)*

And bellow a more complete list of functions, with the link to their full online documentation. This list is the result of an automatic mining in the most important modules and classes of TADbit using the script https://github.com/3DGenomes/TADbit/blob/master/doc/summarize_doc.py.

Root module

- get_dependencies_version: Check versions of TADbit and all dependencies, as well and retrieves system info. May be used to ensure reproducibility.

Alignment module

- generate_shuffle_tads: Returns a shuffle version of a given list of TADs
- randomization_test: Return the probability that original alignment is better than an alignment of randomized boundaries.
- generate_rnd_tads: Generates random TADs over a chromosome of a given size according to a given distribution of lengths of TADs.

TAD class

Specific class of TADs, used only within Alignment objects. It is directly inheriting from python dict. a TAD these keys:

- 'start': position of the TAD
- 'end': position of the TAD
- 'score': of the prediction of boundary
- 'brk': same as 'end'
- 'pos': in the alignment (column number)
- 'exp': Experiment this TAD belongs to
- 'index': of this TAD within all TADs in the Experiment

Alignment class

Alignment of TAD borders

- draw [1]: Draw alignments as a plot.
- get_column: Get a list of column responding to a given characteristic.
- itercolumns: Iterate over columns in the alignment
- iteritems: Iterate over experiment names and aligned boundaries
- itervalues: Iterate over experiment names and aligned boundaries
- write_alignment: Print alignment of TAD boundaries between different experiments. Alignments are displayed with colors according to the TADbit confidence score for each boundary.

Boundary_aligner aligner module

- consensusize: Given two alignments returns a consensus alignment. Used for the generation of multiple alignments
- align: Align Topologically Associating Domain borders. Supports multiple-alignment by building a consensus TAD sequence and aligning each experiment to it.

Boundary_aligner globally module

- needleman_wunsch: Align two lists of TAD boundaries using a Needleman-Wunsh implementation

Boundary_aligner reciprocally module

- find_closest_reciprocal: Function to check the needleman_wunsch algorithm.
- reciprocal: Method based on reciprocal closest boundaries (bd). bd1 will be aligned with bd2 (closest boundary from bd1) if and only if bd1 is the closest boundary of bd2 too (and of course if the distance between bd1 and bd2 is lower than max_dist).

Chromosome module

- load_chromosome: Load a Chromosome object from a file. A Chromosome object can be saved with the save_chromosome function.

ChromosomeSize class

Simple class inheriting from integer designed to hold chromosome size in base pairs

ExperimentList class

Inherited from python built in list, modified for TADbit Experiment.

Mainly, getitem, setitem, and append were modified in order to be able to search for experiments by index or by name, and to add experiments simply using Chromosome.experiments.append(Experiment).

The whole ExperimentList object is linked to a Chromosome instance (Chromosome).

AlignmentDict class

dict of Alignment

Modified getitem, setitem, and append in order to be able to search alignments by index or by name.

linked to a Chromosome

RelativeChromosomeSize class

Relative Chromosome size in base pairs.

Only used for TAD alignment randomization.

Chromosome class

A Chromosome object designed to deal with Topologically Associating Domains predictions from different experiments, in different cell types for a given chromosome of DNA, and to compare them.

- add_experiment: Add a Hi-C experiment to Chromosome
- align_experiments: Align the predicted boundaries of two different experiments. The resulting alignment will be stored in the self.experiment list.
- find_tad: Call the tadbit function to calculate the position of Topologically Associated Domain boundaries
- get_experiment: Fetch an Experiment according to its name. This can also be done directly with Chromosome.experiments[name].
- get_tad_hic: Retrieve the Hi-C data matrix corresponding to a given TAD.
- iter_tads: Iterate over the TADs corresponding to a given experiment.
- save_chromosome: Save a Chromosome object to a file (it uses load from the cPickle). Once saved, the object can be loaded with load_chromosome.
- set_max_tad_size: Change the maximum size allowed for TADs. It also applies to the computed experiments.
- tad_density_plot [1]: Draw an summary of the TAD found in a given experiment and their density in terms of relative Hi-C interaction count.
- visualize [1]: Visualize the matrix of Hi-C interactions of a given experiment

Experiment module

- load_experiment_from_reads: Loads an experiment object from TADbit-generated read files, that are lists of pairs of reads mapped to a reference genome.

Experiment class

Hi-C experiment.

- filter_columns [1]: Call filtering function, to remove artifactual columns in a given Hi-C matrix. This function will detect columns with very low interaction counts; columns passing through a cell with no interaction in the diagonal; and columns with NaN values (in this case NaN will be

replaced by zero in the original Hi-C data matrix). Filtered out columns will be stored in the dictionary Experiment._zeros.

- get_hic_matrix: Return the Hi-C matrix.
- get_hic_zscores: Normalize the Hi-C raw data. The result will be stored into the private Experiment._zscore list.
- load_hic_data: Add a Hi-C experiment to the Chromosome object.
- load_norm_data: Add a normalized Hi-C experiment to the Chromosome object.
- load_tad_def: Add the Topologically Associated Domains definition detection to Slice
- model_region [2]: Generates of three-dimensional models using IMP, for a given segment of chromosome.
- normalize_hic: Normalize the Hi-C data. This normalization step does the same of the tadbit function (default parameters), It fills the Experiment.norm variable with the Hi-C values divided by the calculated weight. The weight of a given cell in column i and row j corresponds to the square root of the product of the sum of column i by the sum of row j. normalization is done according to this formula:
- optimal_imp_parameters [2]: Find the optimal set of parameters to be used for the 3D modeling in IMP.
- print_hic_matrix: Return the Hi-C matrix as string
- set_resolution: Set a new value for the resolution. Copy the original data into Experiment._ori_hic and replace the Experiment.hic_data with the data corresponding to new data (compare_condition).
- view [1]: Visualize the matrix of Hi-C interactions
- write_interaction_pairs: Creates a tab separated file with all the pairwise interactions.
- write_json: Save hic matrix in the json format, read by TADkit.
- write_tad_borders [2]: Print a table summarizing the TADs found by tadbit. This function outputs something similar to the R function.

Hic_data module

HiC_data class

This may also hold the print/write-to-file matrix functions

- add_sections: Add genomic coordinate to HiC_data object by getting them from a fasta file containing chromosome sequences. Orders matters.
- add_sections_from_fasta: Add genomic coordinate to HiC_data object by getting them from a FASTA file containing chromosome sequences
- cis_trans_ratio: Counts the number of interactions occurring within chromosomes (cis) with respect to the total number of interactions
- find_compartments [1] [2]: Search for A/B compartments in each chromosome of the Hi-C matrix. Hi-C matrix is normalized by the number interaction expected at a given distance, and by visibility (one iteration of ICE). A correlation matrix is then calculated from this normalized matrix, and its first eigenvector is used to identify compartments. Changes in sign marking boundaries between compartments. Result is stored as a dictionary of compartment boundaries, keys being chromosome names.
- get_hic_data_as_csr: Returns a scipy sparse matrix in Compressed Sparse Row format of the HiC data in the dictionary
- get_matrix: returns a matrix.

- sum: Sum Hi-C data matrix WARNING: parameters are not meant to be used by external users
- write_compartments [2]: Write compartments to a file.
- write_coord_table: writes a coordinate table to a file.
- write_matrix: writes the matrix to a file.
- yield_matrix: Yields a matrix line by line. Bad row/columns are returned as null row/columns.

Mapping module

- gt_reads: Compare reads accounting for multicontacts
- get_intersection: Merges the two files corresponding to each reads sides. Reads found in both files are merged and written in an output file. Dealing with multiple contacts: - a pairwise contact is created for each possible combnation of the multicontacts. - if no other fragment from this read are mapped than, both are kept - otherwise, they are merged into one longer (as if they were mapped in the positive strand)
- eq_reads: Compare reads accounting for multicontacts
- merge_2d_beds: Merge two result files (file resulting from get_intersection or from the filtering) into one.

Mapping analyze module

- eig_correlate_matrices [1] [2]: Compare the interactions of two Hi-C matrices using their 6 first eigenvectors, with Pearson correlation
- plot_genomic_distribution [1] [2]: Plot the number of reads in bins along the genome (or along a given chromosome).
- plot_strand_bias_by_distance [1]: Classify reads into for categories depending on the strand on which each end is mapped, and plots the proportion of each of these categories in function of the genomic distance between them. The four categories are: - Both read-ends mapped in the forward strand - Both read-ends mapped in the reverse strand - First read-end in the forward strand1, second in the reverse - First read-end in the reverse strand1, second in the forward Note: First/second read-ends are according to their genomic coordinates. The plot is divided in two halves, in order to use different zooms for read-ends mapped very close, and read-ends further (by default the first half goes from a distance of 0 to 2 kb, and the second from 2 kb to 50 kb).
- hic_map [1] [2]: function to retrieve data from HiC-data object. Data can be stored as a square matrix, or drawn using matplotlib
- plot_iterative_mapping [1]: Plots the number of reads mapped at each step of the mapping process (in the case of the iterative mapping, each step is mapping process with a given size of fragments).
- plot_distance_vs_interactions [1]: Plot the number of interactions observed versus the genomic distance between the mapped ends of the read. The slope is expected to be around -1, in logarithmic scale and between 700 kb and 10 Mb (according to the prediction of the fractal globule model).
- correlate_matrices [1] [2]: Compare the interactions of two Hi-C matrices at a given distance, with Spearman rank correlation
- insert_sizes [1]: Plots the distribution of dangling-ends lengths

Mapping filter module

- apply_filter [2]: Create a new file with reads filtered
- filter_reads [2]: Filter mapped pair of reads in order to remove experimental artifacts (e.g. dangling-ends, self-circle, PCR artifacts

Mapping full_mapper module

- full_mapping: Maps FASTQ reads to an indexed reference genome. Mapping can be done either without knowledge of the restriction enzyme used, or for experiments performed without one, like Micro-C (iterative mapping), or using the ligation sites created from the digested ends (fragment-based mapping).
- transform_fastq: Given a FASTQ file it can split it into chunks of a given number of reads, trim each read according to a start/end positions or split them into restriction enzyme fragments

Mapping restriction_enzymes module

- map_re_sites_nochunk: map all restriction enzyme (RE) sites of a given enzyme in a genome. Position of a RE site is defined as the genomic coordinate of the first nucleotide after the first cut (genomic coordinate starts at 1). In the case of HindIII the genomic coordinate is this one: 123456 789
- repaired: returns the resulting sequence after reparation of two digested and repaired ends, marking dangling ends.
- map_re_sites: map all restriction enzyme (RE) sites of a given enzyme in a genome. Position of a RE site is defined as the genomic coordinate of the first nucleotide after the first cut (genomic coordinate starts at 1). In the case of HindIII the genomic coordinate is this one: 123456 789
- religated: returns the resulting sequence after religation of two digested and repaired ends.

Modelling imp_modelling module

- generate_3d_models [2]: This function generates three-dimensional models starting from Hi-C data. The final analysis will be performed on the n_keep top models.

Modelling impmodel module

- load_impmodel_from_xyz: Loads an IMPmodel object using an xyz file of the form:
- load_impmodel_from_cmm: Loads an IMPmodel object using an cmm file of the form:

IMPmodel class

A container for the IMP modeling results.

- objective_function [1]: This function plots the objective function value per each Monte-Carlo step.

Modelling impoptimizer module

IMPoptimizer class

This class optimizes a set of parameters (scale, maxdist, lowfreq and upfreq) in order to maximize the correlation between the models generated by IMP and the input data.

- get_best_parameters_dict:

- load_from_file: Loads the optimized parameters from a file generated with the function: pytadbit.modelling.impoptimizer.IMPoptimizer.write_result. This function does not overwrite the parameters that were already loaded or calculated.
- load_grid_search: Loads one file or a list of files containing pre-calculated Structural Models (keep_models parameter used). And correlate each set of models with real data. Useful to run different correlation on the same data avoiding to re-calculate each time the models.
- plot_2d [1]: A grid of heatmaps representing the result of the optimization.
- plot_3d: A grid of heatmaps representing the result of the optimization.
- run_grid_search [2]: This function calculates the correlation between the models generated by IMP and the input data for the four main IMP parameters (scale, maxdist, lowfreq and upfreq) in the given ranges of values.
- write_result: This function writes a log file of all the values tested for each parameter, and the resulting correlation value. This file can be used to load or merge data a posteriori using the function pytadbit.modelling.impoptimizer.IMPoptimizer.load_from_file

Modelling structuralmodel module

IMPmodel class

A container for the IMP modeling results.

- accessible_surface [1]: Calculates a mesh surface around the model (distance equal to input **radius**) and checks if each point of this mesh could be replaced by an object (i.e. a protein) of a given **radius** Outer part of the model can be excluded from the estimation of accessible surface, as the occupancy outside the model is unknown (see superradius option).
- center_of_mass: Gives the center of mass of a model
- contour: Total length of the model
- cube_side: Calculates the side of a cube containing the model.
- cube_volume: Calculates the volume of a cube containing the model.
- distance: Calculates the distance between one point of the model and an external coordinate
- inaccessible_particles: Gives the number of loci/particles that are accessible to an object (i.e. a protein) of a given size.
- longest_axe: Gives the distance between most distant particles of the model
- min_max_by_axis: Calculates the minimum and maximum coordinates of the model
- persistence_length: Calculates the persistence length (Lp) of given section of the model. Persistence length is calculated according to [Bystricky2004] :
- radius_of_gyration: Calculates the radius of gyration or gyradius of the model Defined as:
- shortest_axe: Minimum distance between two particles in the model
- view_model [1]: Visualize a selected model in the three dimensions. (either with Chimera or through matplotlib).

- write_cmm [2]: Save a model in the cmm format, read by Chimera (http://www.cgl.ucsf.edu/chimera). **Note:** If none of model_num, models or cluster parameter are set, ALL the models will be written.
- write_xyz [2]: Writes a xyz file containing the 3D coordinates of each particle in the model. Outfile is tab separated column with the bead number being the first column, then the genomic coordinate and finally the 3 coordinates X, Y and Z **Note:** If none of model_num, models or cluster parameter are set, ALL the models will be written.

Modelling structuralmodels module

- load_structuralmodels: Loads StructuralModels from a file (generated with save_models).

StructuralModels class

This class contains three-dimensional models generated from a single Hi-C data. They can be reached either by their index (integer representing their rank according to objective function value), or by their IMP random intial number (as string).

- accessibility [1] [2]: Calculates a mesh surface around the model (distance equal to input **radius**) and checks if each point of this mesh could be replaced by an object (i.e. a protein) of a given **radius** Outer part of the model can be excluded from the estimation of accessible surface, as the occupancy outside the model is unkown (see superradius option).
- align_models: Three-dimensional aligner for structural models.
- angle_between_3_particles: Calculates the angle between 3 particles. Given three particles A, B and C, the angle g (angle ACB, shown below):
- average_model: Builds and returns an average model representing a given group of models
- centroid_model: Estimates and returns the centroid model of a given group of models.
- cluster_analysis_dendrogram [1]: Representation of the clustering results. The length of the leaves if proportional to the final objective function value of each model. The branch widths are proportional to the number of models in a given cluster (or group of clusters, if it is an internal branch).
- cluster_models: This function performs a clustering analysis of the generated models based on structural comparison. The result will be stored in StructuralModels.clusters Clustering is done according to a score of pairwise comparison calculated as:
- contact_map [1] [2]: Plots a contact map representing the frequency of interaction (defined by a distance cutoff) between two particles.
- correlate_with_real_data [1]: Plots the result of a correlation between a given group of models and original Hi-C data.
- deconvolve [1]: This function performs a deconvolution analysis of a given froup of models. It first clusters models based on structural comparison (dRMSD), and then, performs a differential contact map between each possible pair of cluster.
- define_best_models: Defines the number of top models (based on the objective function) to keep. If keep_all is set to True in generate_3d_models or in model_region, then the full set of models (n_models parameter) will be used, otherwise only the n_keep models will be available.

- density_plot [1] [2]: Plots the number of nucleotides per nm of chromatin vs the modeled region bins.
- dihedral_angle: Calculates the dihedral angle between 2 planes formed by 5 particles (one common to both planes).
- fetch_model_by_rand_init: Models are stored according to their objective function value (first best), but in order to reproduce a model, we need its initial random number. This method helps to fetch the model corresponding to a given initial random number stored under StructuralModels.models[N]['rand_init'].
- get_contact_matrix: Returns a matrix with the number of interactions observed below a given cutoff distance.
- interactions [1] [2]: Plots, for each particle, the number of interactions (particles closer than the given cut-off). The value given is the average for all models.
- median_3d_dist [1]: Computes the median distance between two particles over a set of models
- model_consistency [1] [2]: Plots the particle consistency, over a given set of models, vs the modeled region bins. The consistency is a measure of the variability (or stability) of the modeled region (the higher the consistency value, the higher stability).
- objective_function_model [1]: This function plots the objective function value per each Monte-Carlo step
- particle_coordinates: Returns the mean coordinate of a given particle in a group of models.
- save_models [2]: Saves all the models in pickle format (python object written to disk).
- view_centroid: shortcut for view_models(tool='plot', show='highlighted', highlight='centroid')
- view_models [1]: Visualize a selected model in the three dimensions (either with Chimera or through matplotlib).
- walking_angle [1] [2]: Plots the angle between successive loci in a given model or set of models. In order to limit the noise of the measure angle is calculated between 3 loci, between each are two other loci. E.g. in the scheme bellow, angle are calculated between loci A, D and G.
- walking_dihedral [1] [2]: Plots the dihedral angle between successive planes. A plane is formed by 3 successive loci.
- zscore_plot [1]: Generate 3 plots. Two heatmaps of the Z-scores used for modeling, one of which is binary showing in red Z-scores higher than upper cut-off; and in blue Z-scores lower than lower cut-off. Last plot is an histogram of the distribution of Z-scores, showing selected regions. Histogram also shows the fit to normal distribution.

Parsers bed_parser module

- parse_bed: simple BED and BEDgraph parser that only checks for the fields 1, 2, 3 and 5 (or 1, 2 and 3 if 5 not availbale).

Parsers genome_parser module

- parse_fasta: Parse a list of fasta files, or just one fasta. WARNING: The order is important

Parsers hic_parser module

- optimal_reader: Reads a matrix generated by TADbit. Can be slower than autoreader, but uses almost a third of the memory
- autoreader: Auto-detect matrix format of HiC data file.
- read_matrix: Read and checks a matrix from a file (using autoreader) or a list.
- is_asymmetric: Helper functions for the autoreader.
- load_hic_data_from_reads:
- symmetrize_dico: Make an HiC_data object symmetric by summing two halves of the matrix
- symmetrize: Make a matrix symmetric by summing two halves of the matrix
- is_asymmetric_dico: Helper functions for the optimal_reader

AutoReadFail class

Exception to handle failed autoreader.

Parsers map_parser module

- parse_map: Parse map files Keep a summary of the results into 2 tab-separated files that will contain 6 columns: read ID, Chromosome, position, strand (either 0 or 1), mapped sequence lebgth, position of the closest upstream RE site, position of the closest downstream RE site. The position of reads mapped on reverse strand will be computed from the end of the read (original position + read length - 1)

Parsers sam_parser module

- parse_sam: Parse sam/bam file using pysam tools. Keep a summary of the results into 2 tab-separated files that will contain 6 columns: read ID, Chromosome, position, strand (either 0 or 1), mapped sequence lebgth, position of the closest upstream RE site, position of the closest downstream RE site

Parsers tad_parser module

- parse_tads: Parse a tab separated value file that contains the list of TADs of a given experiment. This file might have been generated whith the print_result_R or with the R binding for tadbit

Tad_clustering tad_cmo module

- virgin_score: Fill a matrix with zeros, except first row and first column filled with multiple values of penalty.
- core_nw_long: Core of the long Needleman-Wunsch algorithm that aligns matrices
- core_nw: Core of the fast Needleman-Wunsch algorithm that aligns matrices
- optimal_cmo: Calculates the optimal contact map overlap between 2 matrices TODO: make the selection of number of eigen vectors automatic or relying on the summed contribution (e.g. select the EVs that sum 80% of the info)

Tadbit module

- tadbit: The TADbit algorithm works on raw chromosome interaction count data. The normalization is neither necessary nor recommended, since the data is

assumed to be discrete counts. TADbit is a breakpoint detection algorithm that returns the optimal segmentation of the chromosome under BIC-penalized likelihood. The model assumes that counts have a Poisson distribution and that the expected value of the counts decreases like a power-law with the linear distance on the chromosome. This expected value of the counts at position (i,j) is corrected by the counts at diagonal positions (i,i) and (j,j). This normalizes for different restriction enzyme site densities and 'mappability' of the reads in case a bin contains repeated regions.
- batch_tadbit [2]: Use tadbit on directories of data files. All files in the specified directory will be considered data file. The presence of non data files will cause the function to either crash or produce aberrant results. Each file has to contain the data for a single unit/chromosome. The files can be separated in sub-directories corresponding to single experiments or any other organization. Data files that should be considered replicates have to start with the same characters, until the character sep. For instance, all replicates of the unit 'chr1' should start with 'chr1_', using the default value of sep. The data files are read through read.delim. You can pass options to read.delim through the list read_options. For instance if the files have no header, use read_options=list(header=FALSE) and if they also have row names, read_options=list(header=FALSE, row.names=1). Other arguments such as max_size, n_CPU and verbose are passed to tadbit. NOTE: only used externally, not from Chromosome

Utils extraviews module

- compare_models: Plots the difference of contact maps of two group of structural models.
- plot_3d_model [1]: Given a 3 lists of coordinates (x, y, z) plots a three-dimentional model using matplotlib
- color_residues: Function to color residues from blue to red.
- plot_2d_optimization_result [1]: A grid of heatmaps representing the result of the optimization.
- colorize: Colorize with ANSII colors a string for printing in shell. this acording to a given number between 0 and 10
- tad_border_coloring: Colors TAD borders from blue to red (bad to good score). TAD are displayed in scale of grey, from light to dark grey (first to last particle in the TAD)
- tad_coloring: Colors TADs from blue to red (first to last TAD). TAD borders are displayed in scale of grey, from light to dark grey (again first to last border)
- augmented_dendrogram [1]:
- chimera_view [1]: Open a list of .cmm files with Chimera (http://www.cgl.ucsf.edu/chimera) to view models.
- plot_3d_optimization_result: Displays a three dimensional scatter plot representing the result of the optimization.
- nicer: writes resolution number for human beings.

Utils fastq_utils module

- count_reads_approx: Get the approximate number of reads in a FASTQ file. By averaging the sizes of a given sample od randomly selected reads, and relating this mean to the size of the file.
- quality_plot [1]: Plots the sequencing quality of a given FASTQ file. If a restrinction enzyme (RE) name is provided, can also represent the distribution of digested and undigested RE sites and estimate an expected proportion of dangling-ends. Proportion of dangling-ends is inferred by counting the number

of times a dangling-end site, is found at the beginning of any of the reads (divided by the number of reads).

- count_reads: Count the number of reads in a FASTQ file (can be slow on big files, try count_reads_approx)
- estimate_cardinality: Estimates the number of unique elements in the input set values. from: http://blog.notdot.net/2012/09/Dam-Cool-Algorithms-Cardinality-Estimation Arguments: values: An iterator of hashable elements to estimate the cardinality of. k: The number of bits of hash to use as a bucket number; there will be 2**k buckets.

Utils file_handling module

- magic_open: To read uncompressed zip gzip bzip2 or tar.xx files
- wc: Pythonic way to count lines
- get_free_space_mb: Return folder/drive free space (in bytes) Based on stackoverflow answer: http://stackoverflow.com/questions/51658/cross-platform-space-remaining-on-volume-using-python
- which: stackoverflow: http://stackoverflow.com/questions/377017/test-if-executable-exists-in-python

Utils hic_filtering module

- filter_by_mean [1]: fits the distribution of Hi-C interaction count by column in the matrix to a polynomial. Then searches for the first possible
- hic_filtering_for_modelling [1]: Call filtering function, to remove artifactual columns in a given Hi-C matrix. This function will detect columns with very low interaction counts; and columns with NaN values (in this case NaN will be replaced by zero in the original Hi-C data matrix). Filtered out columns will be stored in the dictionary Experiment._zeros.
- filter_by_zero_count:

Utils hmm module

- best_path: Viterbi algorithm with backpointers
- gaussian_prob: of x to follow the gaussian with given E https://en.wikipedia.org/wiki/Normal_distribution

Utils normalize_hic module

- iterative: Implementation of iterative correction Imakaev 2012
- expected: Computes the expected values by averaging observed interactions at a given distance in a given HiC matrix.

Utils tadmaths module

- mean_none: Calculates the mean of a list of values without taking into account the None
- right_double_mad: Double Median Absolute Deviation: a 'Robust' version of standard deviation. Indices variability of the sample. http://eurekastatistics.com/using-the-median-absolute-deviation-to-find-outliers
- zscore: Calculates the log10, Z-score of a given list of values.
- calinski_harabasz: Implementation of the CH score [CalinskiHarabasz1974], that has shown to be one the most accurate way to compare clustering methods [MilliganCooper1985] [Tibshirani2001]. The CH score is:

- newton_raphson: Newton-Raphson method as defined in: http://www.maths.tcd.ie/~ryan/TeachingArchive/161/teaching/newton-raphson.c.html used to search for the persistence length of a given model.
- mad: Median Absolute Deviation: a "Robust" version of standard deviation. Indices variability of the sample. https://en.wikipedia.org/wiki/Median_absolute_deviation

Interpolate class

Simple linear interpolation, to be used when the one from scipy is not available.

Utils three_dim_stats module

- square_distance: Calculates the square distance between two particles.
- dihedral: Calculates dihedral angle between 4 points in 3D (array with x,y,z)
- generate_circle_points: Returns list of 3d coordinates of points on a circle using the Rodrigues rotation formula. see *Murray, G. (2013). Rotation About an Arbitrary Axis in 3 Dimensions* for details
- mass_center: Transforms coordinates according to the center of mass
- generate_sphere_points: Returns list of 3d coordinates of points on a sphere using the Golden Section Spiral algorithm.
- rotate_among_y_axis: Rotate and object with a list of x, y, z coordinates among its center of mass
- calc_eqv_rmsd: Calculates the RMSD, dRMSD, the number of equivalent positions and a score combining these three measures. The measure are done between a group of models in a one against all manner.
- get_center_of_mass: get the center of mass of a given object with list of x, y, z coordinates
- find_angle_rotation_improve_x: Finds the rotation angle needed to face the longest edge of the molecule
- fast_square_distance: Calculates the square distance between two coordinates.
- angle_between_3_points: Calculates the angle between 3 particles Given three particles A, B and C, the angle g (angle ACB, shown below):

- build_mesh: Main function for the calculation of the accessibility of a model.

[1] *(1, 2, 3, 4, 5, 6, 7, 8, 9, 10, 11, 12, 13, 14, 15, 16, 17, 18, 19, 20, 21, 22, 23, 24, 25, 26, 27, 28, 29, 30, 31, 32, 33, 34, 35, 36, 37, 38, 39)* functions generating plots

[2] *(1, 2, 3, 4, 5, 6, 7, 8, 9, 10, 11, 12, 13, 14, 15, 16, 17, 18, 19, 20, 21, 22, 23, 24)* functions writing text files
