# Supplementary material for: Automatic analysis and 3D-modelling of Hi-C data using TADbit reveals structural features of the fly chromatin colors
Source: PLoS Comput Biol. 2017 Jul 19;13(7):e1005665. doi: 10.1371/journal.pcbi.1005665 (PMC5540598; doi:10.1371/journal.pcbi.1005665)
Supplement: S2 Text — Outline of the TAD detection algorithm implemented in TADbit. (PDF) [file pcbi.1005665.s007.pdf]

S2 Text. TAD border detection from interaction matrices.

TADbit analyzes the contact distribution along the genome and subsequently segments it into its constitutive TADs, with each TAD border corresponding to a vertical slice of the Hi-C interaction matrix. TADbit employs a breakpoint detection algorithm that returns the optimal segmentation of the chromosome under BIC-penalized likelihood. Briefly, The number of interactions between loci *i* and *j* separated by Δ nucleotides is assumed to have a Poisson distribution with parameter $w_{ij}\ exp(\alpha + \beta\,\Delta)$, where α and β are TAD-dependent constants and w_{ij} is the normalization factor for the cell at coordinates (*i,j*) of the Hi-C contact matrix. Breakpoint detection methods were developed to segment time series in uniform blocks. In the case of Hi-C data, the correspondence with times series is not straightforward because the measured signal is two-dimensional. This issue is resolved by considering that a single observation is the vector of interactions of a locus with all other loci, in other words, an observation is a column of the Hi-C matrix. In this view, a TAD defines a vertical slice of the Hi-C matrix. Each cell of this slice belongs to one of three categories: the contacts between the TAD and all upstream loci, the intra-TAD contacts, and the contacts between the TAD and all downstream loci. From there, the algorithm proceeds in two phases. In the first, the log-likelihood of every slice (defined by a start and end position) is computed. If the slice does not cover exactly one TAD, at least one of the three categories described above will be composite, which will cause a misfit. As a result, the total log-likelihood of this slice will be low. If the slice covers exactly one TAD, all three categories will be uniform and the log-likelihood of the slice will be high. The search for the optimal decomposition of the Hi-C matrix is carried out by a dynamic programming algorithm based on the following property: if $L_k(s,e)$ denotes the log-likelihood of the optimal segmentation of the slice (*s,e*) into *k* sub-slices, then

$$L_k(1,e) = \min\,(L_{k-1}(1,h) + L_1(h+1,e)$$

where the minimum is taken over all the values of *h*. This formula allows computing the optimal segmentation recursively.

To assign a border score or strength value, the likelihood of each TAD border in the optimal segmentation is penalized by a value equal to the expected gain in log-likelihood for adding a TAD border after the optimum is reached, and the dynamic programming segmentation is restarted. The whole process is carried out 10 times, and each time a border is on the optimal segmentation, it is penalized by this constant. The strength of a

TAD border is the number of times it was included in the optimal segmentation, and it thus ranges from 1 to 10. TAD borders with a score greater than 5 will be considered “robust”, meaning that they are reproducible among different runs; conversely, TAD borders with a score lower than 5 will be considered “weak”, and are likely to be undetectable in replicates or at other resolutions.
