# Supplementary material for: Automatic analysis and 3D-modelling of Hi-C data using TADbit reveals structural features of the fly chromatin colors
Source: PLoS Comput Biol. 2017 Jul 19;13(7):e1005665. doi: 10.1371/journal.pcbi.1005665 (PMC5540598; doi:10.1371/journal.pcbi.1005665)
Supplement: S3 Text — Description of the similarity measure comparing two models generated with TADbit. (PDF) [file pcbi.1005665.s008.pdf]

S3 Text. Structural similarity score for model clustering.

The S_{mirror} score of a given pair of models i and j is computed as:

$$S_{mirror_{i,j}} = Eqv_{i,j} \times \frac{dRMSD_n/\max(dRMSD)}{RMSD_n/\max(RMSD)}$$

Where $Eqv_{i,j}$ is the number of equivalent positions between two superimposed structures within a specific distance cut-off; $dRMSD_n$ is the normalized (*i.e.,* the dRMSD divided by the maximal dRMSD in all structural comparisons) distance RMSD between two aligned structures; and $RMSD_n$ is the normalized RMSD between two aligned structures. This results in a comparison matrix, consisting of all-against-all S_{mirror} scores, which is then used to resolve structural mirrors (conformations with the same IMP objective function that are mirrors of each other). Next, the comparison matrix is input to the
