# Supplementary material for: Automatic analysis and 3D-modelling of Hi-C data using TADbit reveals structural features of the fly chromatin colors
Source: PLoS Comput Biol. 2017 Jul 19;13(7):e1005665. doi: 10.1371/journal.pcbi.1005665 (PMC5540598; doi:10.1371/journal.pcbi.1005665)
Supplement: S4 Text — Description of the Serra et al 2015 dataset to reproduce the data in this article. (PDF) [file pcbi.1005665.s009.pdf]

S4 Text. Serra et al 2015 dataset

All required data and scripts to entirely reproduce the Serra et al 2015 dataset can be downloaded at http://www.3DGenomes.org/datasets/serra_etal (file size: ~300Mb). After you install the necessary software for TADbit to run (see details here: https://github.com/3DGenomes/TADbit) as well as the specific software for the dataset (see below), simply run the script "*0_run_all.sh*". That script will run all necessary sub-scripts that will execute all the steps for reproducing the Serra et al 2015 dataset. Be aware that the scripts are tested in a Linux Box with 24 CPUs and the final output will require a total of 205Gb of disk space of and will run for about 3 days using 20 CPUs.

Other dependencies for reproducing the Serra et al 2015 Dataset:

- wget (http://www.gnu.org/software/wget/) to download the SRA files with the reads from the Hou et al 2012 experiment.
- fastq-dump program from the SRA-toolkit (http://www.ncbi.nlm.nih.gov/books/NBK158900/) to re-format the SRA into FASTQ files.
- All dependencies of TADbit, including GEM (http://algorithms.cnag.cat/wiki/The_GEM_library) and IMP (http://www.integrativemodeling.org).
